# Eligibility for sacubitril/valsartan in heart failure across the ejection fraction spectrum: real‐world data from the Swedish Heart Failure Registry

**DOI:** 10.1111/joim.13165

**Published:** 2020-09-01

**Authors:** G. Savarese, C. Hage, L. Benson, B. Schrage, T. Thorvaldsen, A. Lundberg, M. Fudim, C. Linde, U. Dahlström, G. M. C. Rosano, L. H. Lund

**Affiliations:** ^1^ From the Division of Cardiology Department of Medicine Karolinska Institutet Stockholm Sweden; ^2^ Department of Cardiology University Heart and Vascular Center Hamburg Hamburg Germany; ^3^ Novartis Sverige Kista Sweden; ^4^ Duke University Medical Center Durham NC USA; ^5^ Department of Cardiology and Department of Health, Medicine and Caring Sciences Linkoping University Linkoping Sweden; ^6^ Department of Medical Sciences IRCCS San Raffaele Rome Italy; ^7^ Cardiology Clinical Academic Group St George's Hospitals NHS Trust University of London London UK

**Keywords:** eligibility, PARADIGM‐HF, PARAGON‐HF, sacubitril/valsartan, trial

## Abstract

**Background:**

Randomized controlled trials (RCT) generalizability may be limited due to strict patient selection.

**Objective:**

In a real‐world heart failure (HF) population, we assessed eligibility for sacubitril/valsartan based on PARADIGM‐HF (sacubitril/valsartan effective)/PARAGON‐HF [sacubitril/valsartan effective in mildly reduced ejection fraction (EF)].

**Methods:**

Outpatients from the Swedish HF Registry (SwedeHF) were analysed. In SwedeHF, EF is recorded as <30, 30–39, 40–49 and ≥50%. In PARAGON‐HF, sacubitril/valsartan was effective with EF ≤ 57% (i.e. median). We defined reduced EF/PARADIGM‐HF as EF < 40%, mildly reduced EF/PARAGON‐HF ≤ median as EF 40–49%, and normal EF/PARAGON‐HF > median as EF ≥ 50%. We assessed 2 scenarios: (i) criteria likely to influence treatment decisions (pragmatic scenario); (ii) all criteria (literal scenario).

**Results:**

Of 37 790 outpatients, 57% had EF < 40%, 24% EF 40–49% and 19% EF ≥ 50%. In the pragmatic scenario, 63% were eligible in EF < 50% (67% for EF < 40% and 52% for 40–49%) and 52% in EF ≥ 40% (52% for EF ≥ 50%). For the literal scenario, 32% were eligible in EF < 50% (38% of EF < 40%, 20% of EF 40–49%) and 22% in EF ≥ 40% (25% for EF ≥ 50%). Eligible vs. noneligible patients had more severe HF, more comorbidities and overall worse outcomes.

**Conclusion:**

In a real‐world HF outpatient cohort, 81% of patients had EF < 50%, with 63% eligible for sacubitril/valsartan based on pragmatic criteria and 32% eligible based on literal trial criteria. Similar eligibility was observed for EF 40–49% and ≥50%, suggesting that our estimates for EF < 50% may be reproduced whether or not a higher cut‐off for EF is considered.

## Graphical abstract

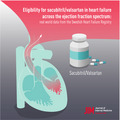

## Introduction

The prognosis in patients with heart failure (HF) remains poor regardless of ejection fraction (EF) [1]. An increasing number of pharmacological treatments have been shown to benefit patients with HF with reduced EF (HFrEF; EF < 40%) [2]. In contrast, randomized controlled trials (RCTs) in patients with HF with preserved EF (HFpEF; EF ≥ 50%) were not successful to date. Thus, treatment recommendations in HFpEF are limited to risk factors modification, comorbidities and symptoms management [2]. Notably, patients with HF with mid‐range EF (HFmrEF; EF = 40–49%) have mildly reduced EF but were excluded from HFrEF trials [1]. Post hoc analyses of RCTs suggest that HFmrEF may benefit from established HFrEF treatments [3].

In the PARADIGM‐HF trial, sacubitril/valsartan improved survival/morbidity in HFrEF compared to enalapril [4]. Recently, the PARAGON‐HF trial tested sacubitril/valsartan vs. valsartan in HF with EF ≥ 45% [5]. Lower, although not statistically significant, rates of cardiovascular death/total HF hospitalizations were observed in patients treated with sacubitril/valsartan [5]. Notably, a prespecified subgroup analysis suggested a potential efficacy in patients with EF ≤ median, which was 57%. This suggests that the benefit of sacubitril/valsartan observed in PARADIGM‐HF could extend at least to the mildly reduced EF [5], and thus may even challenge the current definition of HFrEF to include also the mid‐range or mildly reduced EF.

RCTs apply inclusion/exclusion criteria to ensure HF diagnosis, enrich for modifiable events and minimize nonmodifiable competing risk [6, 7]. Therefore, generalizability of RCTs is often questioned and may limit implementation [8]. We assessed the proportion and characteristics of HF patients with reduced/mildly reduced EF (<50%) and with mildly reduced/preserved EF (EF ≥ 40%) who would be eligible for sacubitril/valsartan based on PARADIGM‐HF/PARAGON‐HF trial inclusion/exclusion criteria.

## Methods

### Data sources

The Swedish Heart Failure Registry (SwedeHF; www.SwedeHF.se) has been previously described [9]. Briefly, patients with clinician‐judged HF have been included in the registry since 11 May 2000. Approximately 80 variables are recorded at discharge from the hospital or after an outpatient clinic visit and entered into a web‐based case report form and database managed by Uppsala Clinical Research Center (www.ucr.se). Echocardiographic parameters other than EF are not collected.

The Swedish Board of Health and Welfare (www.socialstyrelsen.se) administers the National Patient Registry, which provided additional data on baseline comorbidities and the outcome HF hospitalization, and the Cause of Death registry which provided the underlying rather than the immediate cause of death, defined by ICD‐10 codes. ICD‐10 coding in Sweden has been validated, with a positive predictive value ranging between 85% and 95% for most diagnoses. From Statistics Sweden (www.scb.se) we obtained socioeconomic characteristics. All Swedish citizens have unique personal identification numbers that allow linking of disease‐specific health registries and governmental health and statistical registries. The SwedeHF registry and the present analysis with linking of the above registries were approved by an ethics committee. Individual patient consent was not required, but patients were informed of entry into national registries and allowed to opt out.

### Eligibility for sacubitril/valsartan

In SwedeHF, EF is categorized as <30%, 30–39%, 40–49% and ≥50%. In PARAGON‐HF, sacubitril/valsartan was effective with EF ≤ median (57%) [5]. Thus, in this analysis, HFrEF/PARADIGM‐HF was defined as EF < 40%, mildly reduced EF/PARAGON‐HF ≤ median was conservatively defined as EF 40–49% (since we could not define it as ≤57%) and preserved EF/PARAGON‐HF > median was defined as EF ≥ 50%.

Two cohorts were analysed. We were primarily interested in assessing eligibility in patients where sacubitril/valsartan was effective and may be used. Therefore cohort 1 included reduced + mildly reduced EF (EF < 50%) and we used selection criteria from PARADIGM‐HF for EF < 40% and from PARAGON‐HF for EF 40‐49%. Even though clinical trial data do not conclusively support the use of sacubitril/valsartan in strictly preserved EF, we nevertheless wished to also assess eligibility in a PARAGON‐HF like cohort. Therefore, cohort 2 had mildly reduced + preserved EF (EF ≥ 40%) and we used selection criteria from PARAGON‐HF. These cohorts were thus not mutually exclusive (i.e. EF 40–50% was included in both).

SwedeHF patients were considered potentially eligible, and thus included in the denominator for the calculation, if they were outpatients and had EF < 50% (cohort 1) or EF ≥ 40% (cohort 2). If the same patient had multiple registrations, the last one without missing data for location (i.e. inpatient vs. outpatient) and EF was considered.

We considered 2 potential scenarios, the pragmatic and the literal. In the pragmatic scenario eligibility was defined according to selected PARADIGM‐HF/PARAGON‐HF inclusion/exclusion criteria which may be more likely to influence the likelihood of receiving sacubitril/valsartan in clinical practice [age, New York Heart Association (NYHA) II‐IV, N‐terminal pro‐B‐type natriuretic peptide (NT‐proBNP) criteria, history of angioedema, pulmonary arterial hypertension (PARAGON‐HF only), systolic blood pressure (SBP) < 110 mmHg for PARAGON‐HF and <100 mmHg for PARADIGM‐HF, pericardial constriction/hypertrophic/infiltrative cardiomyopathy for PARAGON‐HF only, psychiatric illness for PARAGON‐HF only and estimated glomerular filtration rate (eGFR)<30 mL min^−1^/1.73 m^2^]. In the literal scenario eligibility was defined according to all the trials’ inclusion/exclusion criteria which could be assessed in our dataset. Eligibility was also assessed separately in females vs. males. The original definitions of the trials’ entry criteria [10, 11], as well as the corresponding definitions in SwedeHF are reported Table S1 and in Table 1, respectively.

**Table 1:** Eligibility for sacubitril/valsartan based on PARADIGM‐HF/PARAGON‐HF selection criteria in the main study population (missing data imputed)

		Cohort 1 EF < 50%	Cohort 2 EF ≥ 40%
	No of patients (%)	30618 (81%)	16306 (43%)
*Inclusion criteria*			
1	Written informed consent must be obtained before any assessment is performed	Assumed 100%	Assumed 100%
**2**	**Age ≥ 50 in PARAGON‐HF/≥18 in PARADIGM‐HF**	**98.4%**	**95.4%**
3	EF	100%	100%
4	Diuretic treatment at discharge for PARAGON‐HF/ACEi or ARB equivalent to enalapril 10 mg day^−1^ and BBl at discharge for PARADIGM‐HF	69.8%	69.7%
**5**	**NYHA II‐IV**	**87.9%**	**83.7%**
6	Structural heart disease for PARAGON‐HF	Assumed 100%	Assumed 100%
**7**	**NT‐proBNP criteria**	**84.8%**	**80.4%**
	Eligible (literal scenario) only inclusion criteria	54.4%	54.7%
	Eligible (pragmatic scenario) only inclusion criteria	75.9%	68.6%
*Exclusion criteria*			
1	Any prior echocardiographic measurement of EF < 40% for PARAGON‐HF	96.0%	88.6%
2	Acute coronary syndrome/major cardiovascular interventions within 3 months prior to the index date	89.4%	91.9%
3	Any clinical event within the 6 months prior to visit 1 that could have reduced the EF (PARAGON‐HF)	Assumed 100%	Assumed 100%
4	Current acute decompensated HF	100%	100%
5	Patients with both ACEi and ARB at discharge	97.8%	98.6%
6	History of hypersensitivity to any of the study drugs	Assumed 100%	Assumed 100%
**7**	**Known history of angioedema**	**100%**	**100%**
**8**	**Pulmonary arterial hypertension (included in the pragmatic scenario only for PARAGON‐HF)**	**99.7%**	**99.4%**
8	BMI > 40 kg m^−2^	99.2%	96.7%
8	Haemoglobin < 10 g dL^−1^	99.5%	97.9%
9	SBP ≥ 180 mmHg (PARAGON‐HF)	99.5%	98.1%
9	SBP > 150 mmHg and <180 mmHg at baseline, unless the patient is receiving 3 or more antihypertensive drugs (assuming all receive CCBs)	99.8%	99.1%
**9**	**SBP < 110 mmHg (PARAGON‐HF)/<100 mmHg (PARADIGM‐HF)**	**89.8%**	**86.6%**
10	Use of other investigational drugs	Assumed 100%	Assumed 100%
11	History of dilated cardiomyopathy	97.4%	92.7%
12	Evidence of right‐sided HF in the absence of left‐sided structural heart disease	Assumed 100%	Assumed 100%
**13**	**Pericardial constriction/hypertrophic or infiltrative cardiomyopathy (PARAGON‐HF)**	**98.7%**	**95.0%**
14	Clinically significant congenital heart disease	99.6%	98.7%
15	Hemodynamically significant valvular disease	Assumed 100%	Assumed 100%
16	Stroke or TIA within 3 months prior to index date	98.7%	99.1%
17	Coronary or carotid or valvular heart disease requiring intervention	Assumed 100%	Assumed 100%
18	AF and HR > 110	99.7%	99.0%
19	CRT	94.2%	98.2%
20	Previous major transplant	99.8%	99.5%
21	Trial scenario – Mental disorders (PARAGON‐HF)	99.2%	97.3%
**21**	**Pragmatic scenario – Mental disorders (PARAGON‐HF)**	**99.9%**	**99.5%**
22	Pancreatic disease within 5 years prior to index date (PARAGON‐HF)/Liver disease within 1 year or Crohn within 1 year or Duodenal or gastric ulcers within 3 months (PARADIGM‐HF)	98.8%	99.9%
23	History of liver disease (PARAGON‐HF)	99.7%	98.8%
**24**	**eGFR < 30 mL min^−1^/1.73 m^2^**	**94.7%**	**94.1%**
25	Presence of known functionally significant bilateral renal artery stenosis	Assumed 100%	Assumed 100%
26	Potassium > 5.2 mmol L^−1^	98.3%	98.4%
27	Life expentance < 3 (PARAGON‐HF)/5 (PARADIGM‐HF) years	Assumed 100%	Assumed 100%
28	Noncompliance	Assumed 100%	Assumed 100%
29	Drug or alcohol abuse within the past 12 months	99.9%	99.5%
30	Persons directly involved in the execution of this protocol	Assumed 100%	Assumed 100%
31	Malignancy within the past 5 years prior to index (PARAGON‐HF)	95.5%	84.8%
32	Pregnant or nursing (lactating) women	Assumed 100%	Assumed 100%
33	Women of child‐bearing potential	Assumed 100%	Assumed 100%
	Eligible (literal scenario) only exclusion criteria	58.8%	41.3%
	Eligible (pragmatic scenario) only exclusion criteria	84.1%	77.0%
	Eligible (literal scenario)	32.2%	22.1%
	Eligible (pragmatic scenario)	62.7%	51.7%

Eligibility estimates reported as numbers and percentages and representing the remaining cohort after applying the respective inclusion/exclusion criteria. The criteria were not ordered and mutually exclusive.

Variables included in the pragmatic scenario in bold.

Variables included in the imputation models were: sex, age, follow‐up referral to specialty/primary care, follow‐up referral to heart failure nurse‐led clinic, year of registration, marital status, educational level, income, heart failure duration, New York heart association class, ECG, systolic blood pressure, heart rate, estimate glomerular filtration rate, haemoglobin, N‐terminal pro‐B‐type natriuretic peptide, potassium, angiotensin converting enzyme inhibitors/angiotensin receptor blockers, mineralocorticoid receptor antagonist, diuretic, nitrate, antiplatelet, anticoagulant, statin, beta‐blocker, digoxin, smoking, cardiac resynchronization therapy/implantable cardioverter defibrillator, body mass index category, hypertension, diabetes, ischaemic heart disease, myocardial infarction, peripheral vascular disease, stroke/transient ischaemic attack, valvular disease, dilated cardiomyopathy, chronic obstructive pulmonary disease, malignancies (within 5 years), alcohol/drug abuse (within 1 year), mental disease (within 1 year).

ACEi, Angiotensin converting enzyme inhibitor; AF, atrial fibrillation; ARB, angiotensin receptor blocker; BMI, body mass index; CRT, cardiac resynchronization therapy; eGFR, estimated glomerular filtration rate; HF, heart failure; HFpEF, heart failure with preserved ejection fraction; HR, heart rate; NT‐proBNP, N‐terminal pro‐B‐type natriuretic peptide; NYHA, New York Heart Association; SBP, systolic blood pressure; TIA, Transient Ischaemic Attack.

In order to handle missing data for variables involved in the definition of the inclusion/exclusion criteria, for each scenario we performed 3 different analyses. As main analysis, missing data were imputed. As consistency analysis, (i) patients with missing data for a specific criterion were considered as eligible (missing as eligible); (ii) patients with missing data for any of the inclusion/exclusion criteria were excluded from the analysis (i.e. from both numerator and denominator complete case analysis).

### Statistical analyses

The eligibility estimates were reported as numbers and percentages of the remaining cohort after applying the respective inclusion/exclusion criteria. Baseline characteristics were reported as frequencies (percentages) for categorical variables and as medians [interquartile range (IQR)] for continuous variables and compared in eligible vs. noneligible patients by chi‐square and Mann–Whitney tests, respectively. In the main analysis, missing data were imputed once (since one single value was needed for eligibility calculation)(R‐package mice) using the variables reported in the legend of Table 1. Patients with missing doses of angiotensin converting enzyme inhibitors (ACEi)/angiotensin receptor blocker (ARB) or patients with ACEi/ARB imputed as ‘on treatment’ in EF < 40% analyses (i.e. PARADIGM‐HF criteria) were considered as receiving a dose equivalent to enalapril 10 mg day^−1^.

Incidence rates (per 100 patient‐years) for all‐cause, cardiovascular and noncardiovascular mortality, HF hospitalization and the composite of cardiovascular mortality or HF hospitalization were compared in eligible vs. noneligible patients by exact Poisson test.

A *P*‐value < 0.05 (two‐tailed) was considered statistically significant. All statistical analyses were performed in R software version 3.6.1. The R code for all analysis is available on https://github.com/KIHeartFailure/eligibilitySacubitrilValsartanSwedeHF.

## Results

Between 11 May 2000 and 31 December 2016, 130 420 registrations were recorded in SwedeHF. Of these, 114 737 registrations had entries for EF, with 59 668 registered in outpatient setting. After the exclusion of multiple entries per patient, 37 790 unique registrations/patients were considered for our analyses.

Cohort 1 consisted of 30 618 (81%) patients with mildly reduced/reduced (i.e. EF < 50%), including 21 484 (57%) with reduced EF (EF < 40%) + 9134 (24%) with mildly reduced EF (EF = 40–49%). Cohort 2 consisted of 16 306 (43%) patients with mildly reduced + preserved EF (i.e. EF ≥ 40%), including 9134 (24%) with mildly reduced (EF 40–49%) + 7172 (19%) with preserved EF (EF ≥ 50%). Patients with mildly reduced EF were thus included in both study groups.

Patient characteristics according to EF are reported in Table S2.

### Eligibility for sacubitril/valsartan in cohort 1, that is, patients with reduced + mildly reduced EF (EF < 50%)

**Fig. 1:**
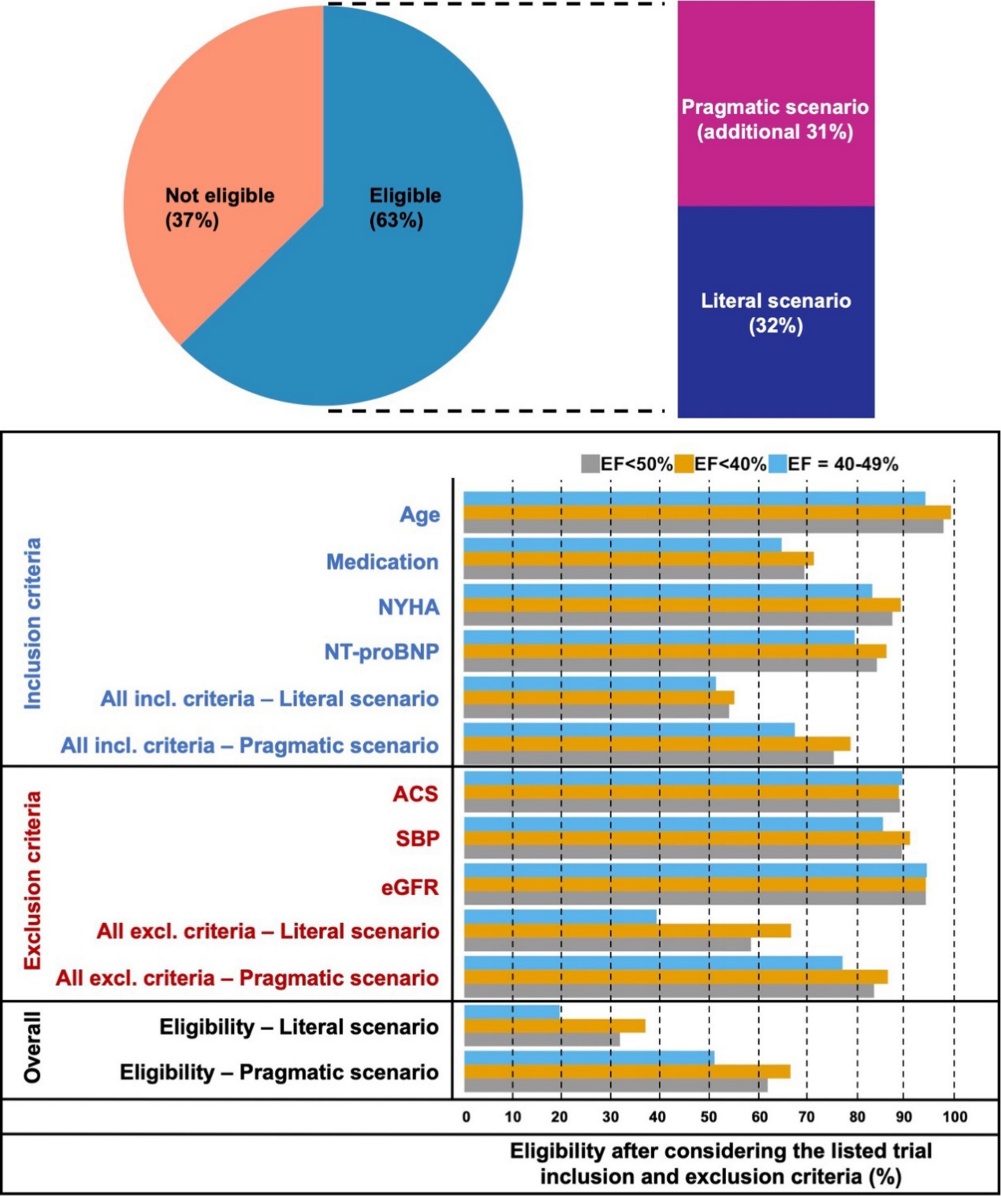
Eligibility for sacubitril/valsartan in heart failure with mildly reduced/reduced ejection fraction (EF < 50%) based on PARADIGM‐HF/PARAGON‐HF selection criteria in the main study population (missing data imputed). Abbreviations as in Table 1.

Of 30 618 patients with EF < 50% screened for eligibility, in the pragmatic scenario, 76% met the inclusion criteria (79% for EF < 40%/PARADIGM‐HF and 68% for EF = 40–49%/PARAGON‐HF), whereas 84% (87% and 78%, respectively) were eligible after applying only the exclusion, and 63% (67% and 52%, respectively) were eligible based on both inclusion and exclusion criteria. Overall eligibility was 64% in females vs. 62% in males. Major unmet inclusion criteria were (i) elevated NT‐proBNP levels and (ii) NYHA class II‐IV. Major exclusion criteria limiting eligibility were (i) hypotension and (ii) renal dysfunction (Table 1; Fig. 1; Table S3).

In the literal scenario, 54% of the population met inclusion criteria (56% for EF < 40%/PARADIGM‐HF and 52% for EF = 40–49%/PARAGON‐HF patients), whereas 59% were eligible after applying only the exclusion criteria (67% and 40%, respectively), with 32% (38% and 20%, respectively), 33% in females vs. 32% in males, meeting both inclusion and exclusion criteria. Major unmet inclusion criteria, which were not considered in the pragmatic scenario, were (i) angiotensin converting enzyme inhibitor (ACEi) or angiotensin receptor blocker (ARB) dose equivalent to enalapril 10mg daily and concomitant use of a beta‐blocker, and (ii) diuretic use. Major exclusion criterion, beyond those considered in the pragmatic scenario, was acute coronary syndrome (ACS) or major cardiovascular surgery within 3 months.

### Eligibility for sacubitril/valsartan based on PARAGON‐HF in cohort 2, that is, patients with mildly reduced + preserved EF (EF ≥ 40%)

Of 16 306 patients with EF ≥ 40%, in the pragmatic scenario 69% met inclusion criteria and 77% were eligible after applying exclusion criteria, leading to an overall eligibility of 52%; 55% in females and 49% in males. Consistently with the findings from cohort 1, major unmet inclusion criteria were (i) elevated NT‐proBNP and (ii) NYHA class II–IV, whereas major exclusion criteria limiting eligibility were (i) hypotension and (ii) renal dysfunction (Table 1; Table S3).

In the literal scenario, 55% fulfilled the inclusion criteria, whereas 41% were eligible after considering only exclusions, leading to an overall eligibility of 22%. Overall eligibility was 26% in females vs. 19% in males. The major unmet inclusion criterion, which was not considered in the pragmatic scenario, was diuretic use. Major exclusion criteria limiting eligibility, beyond those considered in the pragmatic scenario, were (i) malignancies within the last 5 years, (ii) any prior EF < 40% measurement and (iii) ACS or major cardiovascular surgery within 3 months.

In the pragmatic scenario eligibility was 52% in both EF = 40–49% and EF ≥ 50% whereas it was 20% and 25%, respectively, in the literal scenario.

### Consistency analysis

For all the EF groups and scenarios, eligibility was similar when, rather than imputing missing data, we performed complete cases analyses (i.e. patients with and without missing data were not substantially different with regards to eligibility), and as expected, slightly higher when we considered a missing entry as eligible (Table S4).

### Patient characteristics and outcomes in eligible vs. noneligible populations

In the pragmatic scenario (Table 2), regardless of EF, eligible vs. noneligible patients were older, more likely female, less likely to be referred to specialty care, had more severe HF (e.g. more previous HF hospitalizations, higher NYHA class and NT‐proBNP levels) and comorbidities (e.g. more patients had eGFR < 60 vs. ≥60 mL min^−1^/1.73 m^2^, atrial fibrillation, anaemia, hypertension, diabetes, chronic obstructive pulmonary disease, history of cancer). Regardless of EF, use of diuretics, nitrates and digoxin was higher but use of HF devices lower in eligible vs. noneligible patients.

**Table 2:** Baseline characteristics in eligible vs. noneligible patients in the pragmatic scenario

	EF ≥ 50%			EF = 40‐49%			EF < 40%		
	Eligible	Not eligible	*P*‐value	Eligible	Not eligible	*P*‐value	Eligible	Not eligible	*P*‐value
*n*	3710 (51.7)	3462 (48.3)		4720 (51.7)	4414 (48.3)		14474 (67.4)	7010 (32.6)	
*Demographic/Organizational/Socioeconomic Characteristics*									
Sex = Males	1772 (47.8)	1891 (54.6)	<0.001	2968 (62.9)	3012 (68.2)	<0.001	10 563 (73.0)	5212 (74.4)	0.034
Age, median [IQR]	79.0 [73.0, 84.0]	74.0 [64.0, 81.0]	<0.001	77.0 [70.0, 83.0]	70.0 [60.0, 79.0]	<0.001	74.0 [66.0, 80.0]	69.0 [60.0, 77.0]	<0.001
Follow‐up referral = Specialty	1680 (46.4)	1854 (55.4)	<0.001	2718 (59.2)	2890 (67.3)	<0.001	10828 (77.2)	5304 (78.2)	0.092
Follow‐up referral HF nurse‐led clinic	1808 (50.6)	1578 (47.8)	0.019	2465 (54.2)	2242 (52.4)	0.108	8114 (58.3)	3646 (54.3)	<0.001
Year of registration			0.525			<0.001			0.043
2000–2005	160 (4.3)	131 (3.8)		251 (5.3)	162 (3.7)		784 (5.4)	326 (4.7)	
2006–2011	1512 (40.8)	1421 (41.0)		1808 (38.3)	1665 (37.7)		5784 (40.0)	2785 (39.7)	
2012–2016	2038 (54.9)	1910 (55.2)		2661 (56.4)	2587 (58.6)		7906 (54.6)	3899 (55.6)	
Marital status			<0.001			<0.001			<0.001
Married	1705 (46.0)	1680 (48.6)		2415 (51.2)	2328 (52.8)		7434 (51.4)	3603 (51.5)	
Single	859 (23.2)	1040 (30.1)		1226 (26.0)	1456 (33.0)		4652 (32.2)	2508 (35.9)	
Widowed	1144 (30.9)	740 (21.4)		1076 (22.8)	624 (14.2)		2363 (16.4)	883 (12.6)	
Educational level			<0.001			<0.001			<0.001
Compulsory school	1856 (51.1)	1448 (42.6)		2173 (46.7)	1657 (38.2)		6472 (45.5)	2669 (38.8)	
Secondary school	1250 (34.4)	1313 (38.7)		1730 (37.2)	1858 (42.8)		5566 (39.1)	2954 (43.0)	
University	526 (14.5)	636 (18.7)		753 (16.2)	825 (19.0)		2186 (15.4)	1250 (18.2)	
Income			<0.001			<0.001			<0.001
Low	1488 (40.1)	1198 (34.6)		1626 (34.5)	1259 (28.6)		4778 (33.1)	2088 (29.9)	
Medium	1338 (36.1)	1065 (30.8)		1699 (36.0)	1358 (30.8)		4936 (34.2)	2062 (29.5)	
High	882 (23.8)	1197 (34.6)		1392 (29.5)	1791 (40.6)		4735 (32.8)	2844 (40.7)	
*Clinical Characteristics*									
HF duration ≤ 6 months	2281 (62.8)	2274 (66.8)	<0.001	2818 (60.8)	2551 (58.8)	0.054	7937 (55.7)	4042 (58.5)	<0.001
Previous HF hospitalization within 9 months	1115 (30.1)	914 (26.4)	0.001	1292 (27.4)	1047 (23.7)	<0.001	6446 (44.5)	2708 (38.6)	<0.001
Previous HF hospitalization within 12 months	1166 (31.4)	986 (28.5)	0.007	1391 (29.5)	1114 (25.2)	<0.001	6766 (46.7)	2860 (40.8)	<0.001
NYHA class			<0.001			<0.001			<0.001
I	0 (0.0)	978 (34.0)		0 (0.0)	1328 (33.6)		0 (0.0)	2013 (31.5)	
II	1813 (59.3)	1097 (38.2)		2813 (66.7)	1665 (42.1)		7457 (56.4)	2280 (35.7)	
III	1197 (39.2)	740 (25.7)		1357 (32.2)	906 (22.9)		5546 (41.9)	1926 (30.2)	
IV	47 (1.5)	60 (2.1)		46 (1.1)	55 (1.4)		230 (1.7)	168 (2.6)	
ECG			<0.001			<0.001			<0.001
Atrial fibrillation	1596 (44.2)	1224 (36.5)		1769 (38.1)	1330 (30.8)		4993 (35.0)	1517 (22.0)	
PM/other rhythm	313 (8.7)	278 (8.3)		533 (11.5)	388 (9.0)		1785 (12.5)	869 (12.6)	
Sinus rhythm	1700 (47.1)	1853 (55.2)		2338 (50.4)	2607 (60.3)		7491 (52.5)	4500 (65.3)	
Systolic blood pressure	130.0 [120.0, 145.0]	122.5 [107.0, 140.0]	<0.001	130.0 [120.0, 140.0]	120.0 [105.0, 138.0]	<0.001	122.0 [110.0, 140.0]	120.0 [98.0, 135.0]	<0.001
Diastolic blood pressure	74.0 [65.0, 80.0]	70.0 [60.0, 80.0]	<0.001	75.0 [70.0, 80.0]	70.0 [63.0, 80.0]	<0.001	70.0 [65.0, 80.0]	70.0 [60.0, 80.0]	<0.001
Heart rate	70.0 [62.0, 80.0]	69.0 [60.0, 78.0]	0.002	70.0 [60.0, 79.0]	68.0 [60.0, 78.0]	<0.001	70.0 [61.0, 80.0]	68.0 [60.0, 77.0]	<0.001
eGFR			<0.001			<0.001			<0.001
<30 mL min^−1^/1.73 m^2^	0 (0.0)	474 (14.2)		0 (0.0)	458 (10.6)		0 (0.0)	1125 (16.3)	
30–<60 mL min^−1^/1.73 m^2^	2061 (56.8)	1100 (32.9)		2288 (49.3)	1176 (27.2)		6576 (46.2)	1827 (26.4)	
≥60 mL min^−1^/1.73 m^2^	1569 (43.2)	1770 (52.9)		2350 (50.7)	2686 (62.2)		7661 (53.8)	3959 (57.3)	
Haemoglobin (g L^−1^), median [IQR]	130.0 [119.0, 140.0]	133.0 [122.0, 144.0]	<0.001	134.0 [123.0, 144.0]	137.0 [124.0, 148.0]	<0.001	136.0 [125.0, 147.0]	137.0 [125.0, 148.0]	0.001
NT‐proBNP (pg mL^−1^), median [IQR]	1711.0 [976.0, 3200.0]	839.5 [240.0, 2527.0]	<0.001	1809.0 [978.0, 3342.3]	763.5 [230.3, 2300.0]	<0.001	2630.0 [1388.0, 5171.0]	1124.0 [352.2, 4140.0]	<0.001
Potassium (mEq L^−1^), median [IQR]	4.2 [3.9, 4.5]	4.2 [4.0, 4.5]	0.054	4.2 [4.0, 4.5]	4.2 [4.0, 4.5]	0.792	4.3 [4.0, 4.6]	4.3 [4.0, 4.6]	0.001
BMI (kg m^−2^)			0.418			0.051			0.001
≤30	1409 (68.3)	1291 (66.3)		2010 (72.5)	1820 (70.2)		6615 (75.6)	3036 (72.6)	
30–40	562 (27.2)	563 (28.9)		699 (25.2)	690 (26.6)		1965 (22.4)	1046 (25.0)	
>40	93 (4.5)	93 (4.8)		63 (2.3)	82 (3.2)		174 (2.0)	102 (2.4)	
*Treatments*									
ACEi/ARB	3048 (82.7)	2764 (80.3)	0.010	4219 (90.0)	3918 (89.3)	0.314	13517 (94.2)	6413 (92.2)	<0.001
MRA	1054 (28.6)	1001 (29.0)	0.677	1281 (27.3)	1152 (26.2)	0.278	5702 (39.6)	2653 (38.0)	0.029
Diuretic	3067 (82.9)	2315 (67.2)	<0.001	3482 (74.1)	2451 (55.8)	<0.001	11327 (78.5)	4603 (66.0)	<0.001
Nitrate	549 (14.9)	382 (11.1)	<0.001	631 (13.4)	423 (9.6)	<0.001	1756 (12.2)	749 (10.7)	0.002
Antiplatelet	1337 (36.6)	1169 (34.4)	0.055	2056 (44.1)	1858 (42.7)	0.182	6166 (43.1)	3250 (47.1)	<0.001
Anticoagulant	1914 (51.8)	1538 (44.5)	<0.001	2314 (49.2)	1856 (42.2)	<0.001	7076 (49.0)	2869 (41.1)	<0.001
Statin	1693 (45.8)	1467 (42.4)	0.005	2527 (53.7)	2269 (51.6)	0.040	7460 (51.7)	3809 (54.5)	<0.001
Beta‐blocker	3066 (82.8)	2815 (81.5)	0.154	4194 (89.1)	3841 (87.2)	0.005	13496 (93.4)	6475 (92.6)	0.028
Digoxin	595 (16.1)	403 (11.7)	<0.001	684 (14.5)	466 (10.6)	<0.001	2390 (16.6)	747 (10.7)	<0.001
HF device			0.001			0.035			<0.001
CRT‐P	16 (0.5)	19 (0.6)		64 (1.4)	53 (1.2)		419 (2.9)	211 (3.0)	
CRT‐D	15 (0.4)	23 (0.7)		49 (1.1)	52 (1.2)		563 (3.9)	345 (5.0)	
ICD	20 (0.6)	48 (1.5)		59 (1.3)	88 (2.1)		531 (3.7)	378 (5.5)	
*Comorbidities*									
Anaemia	1216 (34.2)	959 (29.5)	<0.001	1358 (30.2)	1077 (25.8)	<0.001	3899 (28.1)	1793 (26.8)	0.051
Smoking			0.007			0.305			0.741
Current	187 (7.2)	234 (9.4)		366 (10.7)	384 (11.7)		1453 (12.8)	725 (13.2)	
Former	1071 (41.1)	1048 (41.9)		1505 (44.0)	1464 (44.5)		5365 (47.3)	2579 (47.1)	
Never	1351 (51.8)	1218 (48.7)		1551 (45.3)	1442 (43.8)		4521 (39.9)	2171 (39.7)	
Hypertension	2526 (69.3)	1960 (57.7)	<0.001	2812 (60.9)	2033 (46.9)	<0.001	7120 (50.6)	3046 (44.5)	<0.001
Diabetes	972 (26.4)	728 (21.1)	<0.001	1148 (24.4)	866 (19.7)	<0.001	3551 (24.6)	1576 (22.6)	0.001
Ischaemic heart disease	1687 (45.5)	1373 (39.7)	<0.001	2619 (55.5)	2196 (49.8)	<0.001	7709 (53.3)	3778 (53.9)	0.391
Myocardial infarction	769 (20.7)	632 (18.3)	0.009	1436 (30.4)	1279 (29.0)	0.136	4301 (29.7)	2166 (30.9)	0.079
Coronary revascularization	917 (24.7)	697 (20.1)	<0.001	1574 (33.3)	1373 (31.1)	0.023	4701 (32.5)	2347 (33.5)	0.147
Peripheral vascular disease	384 (10.4)	299 (8.6)	0.015	545 (11.5)	367 (8.3)	<0.001	1507 (10.4)	665 (9.5)	0.037
Stroke/TIA	582 (15.7)	418 (12.1)	<0.001	718 (15.2)	494 (11.2)	<0.001	2004 (13.8)	812 (11.6)	<0.001
Atrial fibrillation	2235 (60.8)	1770 (51.5)	<0.001	2566 (54.8)	1980 (45.1)	<0.001	7139 (49.6)	2616 (37.5)	<0.001
Valvular disease	1101 (29.7)	869 (25.1)	<0.001	1122 (23.8)	838 (19.0)	<0.001	2708 (18.7)	1044 (14.9)	<0.001
COPD	583 (15.7)	486 (14.0)	0.050	679 (14.4)	513 (11.6)	<0.001	1993 (13.8)	825 (11.8)	<0.001
Liver disease (within 1 year)	42 (1.1)	52 (1.5)	0.203	48 (1.0)	59 (1.3)	0.186	186 (1.3)	106 (1.5)	0.199
Malignancy (within 5 years)	616 (16.6)	488 (14.1)	0.004	755 (16.0)	617 (14.0)	0.008	2040 (14.1)	886 (12.6)	0.004
Pancreatic disease (within 1 year)	46 (1.2)	54 (1.6)	0.292	52 (1.1)	59 (1.3)	0.353	197 (1.4)	114 (1.6)	0.143
Mental disease (within 1 year)	73 (2.0)	122 (3.5)	<0.001	108 (2.3)	139 (3.1)	0.013	552 (3.8)	259 (3.7)	0.696

ACEi, Angiotensin converting enzyme inhibitor; ARB, angiotensin receptor blocker; BMI, body mass index; COPD: chronic obstructive pulmonary disease; CRT‐D, cardiac resynchronization therapy‐defibrillator; CRT‐P, cardiac resynchronization therapy‐pacemaker; ECG, electrocardiogram; EF, ejection fraction; eGFR, estimated glomerular filtration rate; HF, heart failure; ICD, implantable cardioverter defibrillator; IQR, interquartile range; MRA, mineralocorticoid receptor antagonist; NT‐proBNP, N‐terminal pro‐B‐type natriuretic peptide; NYHA, New York Heart Association; TIA, Transient Ischaemic Attack.

In HFrEF, eligible patients were more likely to receive ACEi/ARB, beta‐blockers and mineralocorticoid receptor antagonists. Ischaemic heart disease was more prevalent in eligible vs. noneligible patients in HFpEF and HFmrEF, with no differences in HFrEF. There were few differences in patient characteristics according to eligibility in the literal vs. pragmatic scenario which reflected the stricter exclusion criteria applied (Table S5).

**Fig. 2:**
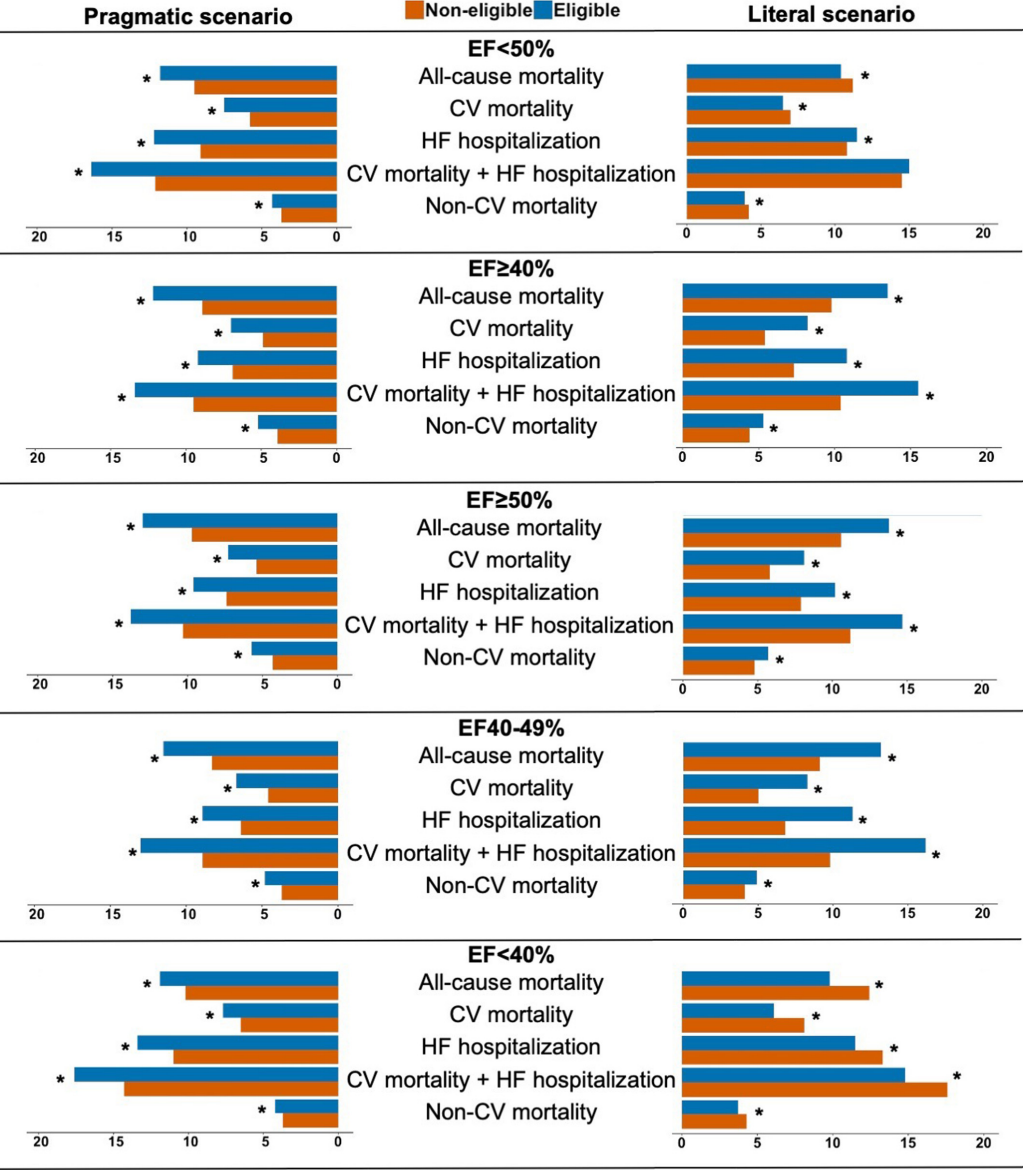
Event rates in eligible vs. noneligible patients. *P‐value < 0.05. Event rates reported as *100 patient‐years. CV, cardiovascular; HF, heart failure; HFrEF, heart failure with reduced ejection fraction; HFpEF, heart failure with preserved ejection fraction.

Event rates for all the outcomes in eligible vs. noneligible patients are reported in Fig. 2 and Table S6 and were higher in eligible patients regardless of EF in the pragmatic scenario. Eligible patients had higher event rates for both cardiovascular, noncardiovascular and all‐cause mortality. Similar results were reported in the literal scenario except for slightly lower mortality rates in eligible vs. ineligible patients with EF < 50% or EF < 40%, and lower HF hospitalization risk in eligible vs. noneligible in EF < 40%.

## Discussion

In a large and contemporary real‐world cohort with HF across the EF spectrum, in a pragmatic scenario eligibility for sacubitril/valsartan was 63% and 52% in EF < 50% and EF ≥ 40%, respectively, whereas the corresponding estimates were 28% and 19% when all the trials inclusion/exclusion criteria were considered (i.e. literal scenario).

Regardless of EF, in the pragmatic scenario, patients were more likely ineligible by not meeting inclusion criteria, mainly due to an insufficiently severe HF status. In the literal scenario, in EF < 50% inclusion and exclusion criteria similarly impacted eligibility, whereas in EF ≥ 40% patients were more likely excluded due to meeting exclusion criteria, mainly related to comorbidities, which is consistent with the increasing comorbidity burden with higher EF.

In PARAGON‐HF sacubitril/valsartan improved mortality/morbidity in EF = 45‐57% and, therefore, its use might possibly be considered in the lower range of HFpEF and for HFmrEF [12]. In our analysis, we could not evaluate eligibility based on the EF = 57% cut‐off since EF is collected as a categorical variable in SwedeHF. However, this specific cut‐off is unlikely to be applied by any stakeholder and even less in clinical practice. Also, we observed overall same eligibility estimates in EF = 40–49% and EF ≥ 50% (52% in both EF groups in the pragmatic scenario and 17% vs. 19%, respectively, in the literal scenario), suggesting similar eligibility whether a higher EF cut‐off is considered or not.

### Major selection criteria driving eligibility

#### Ineligibility due to inclusion criteria

##### Ongoing treatment with ACEi/ARB blockers

Amongst literal inclusion criteria, nonuse of beta‐blockers or ACEi/ARB equivalent to enalapril 10 mg day^−1^ in EF < 50% and nonuse of diuretics at baseline in EF ≥ 40% led to the noneligibility of 30% of our population. In HFrEF, whereas optimal use of ACEi/ARB is still a requirement for initiating sacubitril/valsartan in some of the current guidelines (e.g. European) [2], this is not considered in the regulatory labelling. Additionally, PIONEER‐HF and TRANSITION suggest that sacubitril/valsartan is effective and safe also in ACEi/ARB‐naïve patients [13, 14]. Based on all these considerations, we did not consider previous ACEi/ARB use/dose as a limiting factor for sacubitril/valsartan initiation and thus was not included for our pragmatic scenario.

##### Ongoing treatment with diuretics

PARAGON‐HF included patients with symptoms of HF requiring treatment with diuretics for at least 30 days prior to the screening visit. In our population we could not assess use of diuretics prior to the index visit but we could evaluate use of diuretics at baseline. Based on the discrepancy between our and PARAGON‐HF definition, we did not consider use of diuretics at baseline as part of the pragmatic scenario definition. However, it is likely that most patients not fulfilling this inclusion criterion did also not meet other important inclusion criteria which were instead considered in our pragmatic scenario, that is, were not included due to low NT‐proBNP levels or NYHA class. The gap between eligibility estimates at the pragmatic vs. literal scenario was impacted more by the number of patients meeting the exclusion criteria rather than not meeting the inclusion criteria.

#### Ineligibility due to exclusion criteria

The number of patients ineligible due to the exclusion criteria was higher with increasing EF, which is consistent with the higher burden of comorbidities in HFpEF vs. HFmrEF vs. HFrEF.

##### ACS intervention within 3 months

The exclusion of patients with history of ACS/major cardiovascular interventions within 3 months prior to the index event explained the exclusion of 15–20% of the population across the EF spectrum. However, initiation of sacubitril/valsartan might be simply delayed after an acute ischaemic event or cardiovascular intervention and thus this exclusion criterion was not considered as likely to influence eligibility in real‐world practice.

##### History of cancer

History of cancer within 5 years prior to the enrolment was an exclusion criterion which was met in 20% of our population with EF ≥ 40%, highlighting the considerable prevalence of cancer in HF with mid‐range/preserved EF [15]. However, this trial criterion is meant to reduce competing risk. There is no reason to believe patients with cancer should derive any less symptom relief or in most cases, prognostic benefit and thus was not considered in the pragmatic scenario.

##### Low NYHA class, low eGFR, low NT‐proBNP or hypotension

There were no major differences in the proportion of patients meeting the individual inclusion/exclusion criteria considered in the pragmatic scenario in EF < 50% vs. EF ≥ 40%. In our cohort, around 15‐20% of patients were not included due to asymptomatic HF (NYHA class I) or low NT‐proBNP levels. Severe kidney disease and hypotension limitedly impacted on eligibility estimates regardless of EF, with 6% of our population excluded for eGFR < 30 mL min^−1^/1.73 m^2^ and 87/89% for SBP < 100 mmHg according to PARADIGM‐HF/110 mmHg according to PARAGON‐HF, respectively.

These estimates were similar to those reported in the ESC‐EORP‐HFA HF‐LT registry outpatient cohort based on PARADIGM‐HF selection criteria [16]. Conversely, in a recent study from the GTWG‐HF registry assessing eligibility for sacubitril/valsartan based on PARAGON‐HF selection criteria, 14% of patients with EF > 40% were excluded for eGFR < 30 mL min^−1^/1.73 m^2^ and 29% for SBP < 100 mmHg [17]. Higher estimates in GTWG‐HF vs. SwedeHF population might be explained by the different characteristics of the populations, with our study considering only outpatients and GTWG‐HF enrolling only hospitalized patients (which, incidentally, were not eligible for PARAGON‐HF). Differences in risk profile might also explain the differences in the overall eligibility for sacubitril/valsartan observed in these two cohorts, with 19% eligible according to literal PARAGON‐HF selection criteria in SwedeHF vs. 10% in GTWG‐HF [17].

### Patient characteristics and outcomes according to eligibility status

Regardless of EF, patient characteristics and outcomes amongst eligible patients were linked to more severe HF and overall heavier burden of comorbidities. This was surprising since randomized trials exclude patients with multiple comorbidities, which is suggested to limit generalizability to real‐world care. Similar patterns were shown in a previous SwedeHF analysis, assessing eligibility in HFrEF based on PARADIGM‐HF selection criteria [18]. These findings may be explained by PARADIGM‐HF/PARAGON‐HF inclusion criteria being adequately defined to identify a high‐risk HF outpatient population and, at the same time, by trial exclusion criteria only leading to the exclusion of patients with very specific characteristics which were not linked with multiple comorbidities and more severe HF. This hypothesis might be supported by the fact that although exclusion criteria were many and thus impacted overall eligibility, most of them individually contributed with the exclusion of a limited proportion of patients. Conversely, in the GTWG‐HF PARAGON‐HF analysis, eligible patients had generally lower rates of comorbid diseases and overall lower 1‐year mortality compared to those who were noneligible [17]. Applying the selection criteria of PARAGON‐HF, which has been designed to enrol chronic HF patients, to a very high‐risk population, such as the inpatient cohort of GTWG‐HF, likely excluded patients with less severe or chronic stable HF. Since cardiovascular trials use inclusion/exclusion criteria to attempt to select patients with higher cardiovascular risk and lower noncardiovascular competing risk, one may expect that eligible patients have higher cardiovascular outcome event rates than noneligible, but that for noncardiovascular outcomes eligible have lower event rates than noneligible. Therefore, our observation of greater both cardiovascular, noncardiovascular and all‐cause mortality in eligible vs. noneligible patients, suggests that designing trials to maximize modifiable cardiovascular risk and minimize nonmodifiable noncardiovascular risk remains difficult, not only in HFpEF but also in HFrEF.

### Limitations

Eligibility was assessed cross‐sectionally and may change over time reflecting the natural course of HF. Definitions of some inclusion/exclusion criteria were slightly different from the trials due to the available variables and EF categories collected in the SwedeHF registry. As our analysis was based on a national quality registry with incomplete coverage (54% in inpatient setting) and patients enrolled in SwedeHF have been shown to be younger, have less comorbidities and to receive better care compared to those who are not in the registry, generalizability of the results might be somehow overestimated [19]. Finally, several criteria could not be applied due to lack of required information in the registry, which might have led to overestimated eligibility, in particular in the literal scenario.

## Conclusions

In a real‐world outpatient HF cohort, 81% of the patients had EF < 50%, with 63% eligible for sacubitril/valsartan based on pragmatic criteria and 29% based on literal trial criteria. Similar eligibility was observed for EF 40–49% and ≥50%, suggesting that our eligibility estimates for EF < 50% may be reproduced whether a higher cut‐off for EF is considered or not. Our data may help these multiple stakeholders to assess the implications of recommendations and decisions and may help future trial design by estimating consequences of particular inclusion/exclusion criteria decisions. Ultimately, the use of sacubitril/valsartan may be affected by our study as well as multiple additional factors, such as interpretation of the trials, label, guidelines, payor criteria and comprehensive clinician assessment in individual patient cases.

## Funding

This study has been supported by Novartis. LHL is supported by the Swedish Research Council [grants 2013‐23897‐104604‐23 and 523‐2014‐2336] and the Swedish Heart Lung Foundation [grants 20120321 and 20150557].

## Conflicts of interest

GS reports grants and personal fees from Vifor, non‐financial support from Boehringer Ingelheim, personal fees from Societa´ Prodotti Antibiotici, grants from MSD, grants and personal fees from AstraZeneca, personal fees from Roche, personal fees from Servier, personal fees from Medtronic, personal fees from Cytokinetics, grants from Novartis, personal fees from GENESIS, grants from Boston Scientific. LHL reports personal fees from Merck, personal fees from Sanofi, grants and personal fees from Vifor‐Fresenius, grants and personal fees from AstraZeneca, grants and personal fees from Relypsa, personal fees from Bayer, grants from Boston Scientific, grants and personal fees from Novartis, personal fees from Pharmacosmos, personal fees from Abbott, grants and personal fees from Mundipharma, personal fees from Medscape, personal fees from Myokardia, grants and personal fees from Boehringer Ingelheim, outside the submitted work. CL, GMCR, CH, TT and LB have nothing to disclose. MF reports personal fees from Daxor, personal fees from AxonTherapies, personal fees from Galvani, outside the submitted work. BS reports personal fees from AstraZeneca, outside the submitted work. UD reports grants from AstraZeneca, honoraria from AstraZeneca, Boehringer Ingelheim, Amgen, Novartis, Pfizer, outside the submitted work. AL is employed by Novartis Sweden.

## Author Contribution

**Gianluigi Savarese:** Conceptualization (lead); Methodology (lead); Resources (lead); Visualization (equal); Writing‐original draft (equal). **Camilla Hage:** Writing‐review & editing (equal). **lina benson:** Data curation (lead); Formal analysis (lead); Methodology (equal); Writing‐review & editing (equal). **Benedikt Schrage:** Writing‐original draft (equal); Writing‐review & editing (equal). **Tonje Thorvaldsen:** Writing‐review & editing (supporting). **Anna Lundberg:** Conceptualization (supporting); Funding acquisition (equal); Writing‐review & editing (equal). **Marat Fudim:** Writing‐review & editing (equal). **Ulf Dahlström:** Writing‐review & editing (equal). **Giuseppe Rosano:** Writing‐review & editing (equal). **Lars H Lund:** Conceptualization (equal); Writing‐review & editing (equal).

## Supporting information


**Table S1**. PARAGON‐HF and PARADIGM‐HF inclusion/exclusion criteria and corresponding definitions in the Swedish Heart Failure registry.
**Table S2** **.** Baseline characteristics according to the ejection fraction group.
**Table S3** **.** Eligibility for sacubitril/valsartan based on PARADIGM‐HF/PARAGON‐HF selection criteria in the main study population (missing data imputed) by ejection fraction phenotype.
**Table S4** **.** Eligibility for sacubitril/valsartan based on PARADIGM‐HF/PARAGON‐HF selection criteria according to the ejection fraction group: consistency analysis.
**Table S5** **.** Baseline characteristics in eligible vs. non eligible patients in the literal scenario.
**Table S6** **.** Outcome analysis according to eligibility status.
