# Supplementary material for: Eligibility for sacubitril/valsartan in heart failure across the ejection fraction spectrum: real‐world data from the Swedish Heart Failure Registry
Source: J Intern Med. 2020 Sep 1;289(3):369–84. doi: 10.1111/joim.13165 (PMC7984286; doi:10.1111/joim.13165)
Supplement: Supplementary file 1 — Table S1. PARAGON‐HF and PARADIGM‐HF inclusion/exclusion criteria and corresponding definitions in the Swedish Heart Failure registry. Table S2 . Baseline characteristics according to the ejection fraction group. Table S3 . Eligibility for sacubitril/valsartan based on PARADIGM‐HF/PARAGON‐HF selection criteria in the main study population (missing data imputed) by ejection fraction phenotype. Table S4 . Eligibility for sacubitril/valsartan based on PARADIGM‐HF/PARAGON‐HF selection criteria according to the ejection fraction group: consistency analysis. Table S5 . Baseline characteristics in eligible vs. non eligible patients in the literal scenario. Table S6 . Outcome analysis according to eligibility status. [file JOIM-289-369-s001.docx]

**SUPPLEMENTARY MATERIAL**

**Supplementary Table 1. PARAGON-HF and PARADIGM-HF inclusion/exclusion criteria and corresponding definitions in the Swedish Heart Failure registry**

| **Inclusion criteria** | | | | | |
| --- | --- | --- | --- | --- | --- |
|  | **PARAGON-HF (EF≥40%)** | | **PARADIGM-HF (EF<40%)** | | |
| **Criterion** | **Definition** | **Source and codes** | **Criterion** | **Definition** | **Sources and codes** |
| ﻿1. Written informed consent must be obtained before any assessment is performed | -- | -- | Able to give written informed consent | -- | -- |
| **﻿2. ≥50 years of age, male or female** | **As in PARAGON** | **SwedeHF variable** | **age 18 years or older** | **As in PARADIGM** | **SwedeHF variable** |
| ﻿3. EF ≥45% by echocardiography during the screening epoch, or within 6 months prior to screening visit (any local EF measurement made using echocardiography only) | The study population consisted of patients with EF ≥ 40% at baseline. | -- | EF ≤35% (initially this was ≤40% but changed in a protocol  amendment dated 15 December 2010) | The study population consisted of patients with EF <40% at baseline | -- |
| ﻿﻿4. Symptom(s) of HF requiring treatment with diuretic(s) for at least 30 days prior to screening visit | Diuretic treatment at baseline | SwedeHF variable | - Treatment with a stable dose of an ACEi or an ARB equivalent to enalapril 10 mg/day for at least 4  weeks before the screening visit; AND  - Treatment with a stable dose of a beta-blocker for at least 4 weeks prior to the screening visit, unless contraindicated or not tolerated. | - ACEi or an ARB equivalent to enalapril 10 mg/day at the baseline; AND  - beta-blocker at the baseline | SwedeHF variable |
| **5. Current symptom(s) of HF (NYHA functional class II to IV) at screening visit** | **NYHA II-IV at baseline** | **SwedeHF variable** | **NYHA functional class II–IV** | **NYHA II-IV at baseline** | **SwedeHF variable** |
| 6. Structural heart disease evidenced by at least 1 of the following echocardiography findings (any local measurement made during the screening epoch or within the 6 months prior to screening visit): a) LA enlargement defined by at least 1 of the following: LA width (diameter) ≥3.8 cm or LA length ≥5.0 cm or LA area ≥20 cm2 or LA volume ≥55 ml or LA volume index ≥29 ml/m2  b) LVH defined by septal thickness or posterior wall thickness ≥1.1 cm | -- | -- | -- | -- | -- |
| **7. Patients with at least 1 of the following: a) HF hospitalization (defined as HF listed as the major reason for hospitalization) within 9 months prior to screening visit and NT-proBNP >200 pg/ml for patients not in AF or >600 pg/ml for patients in AF on screening ECG, or**  **b) NT-proBNP >300 pg/ml for patients not in AF or >900 pg/ml for patients in AF on the screening visit ECG** | **As in PARAGON** | **ECG: SwedeHF variable (pacemaker/other rhythm was considered as non-AF).**  **NT-proBNP: SwedeHF variable**  **Prior HF hospitalization: NPR ICD codes: I50, I11.0, I42.0, I42.3-I42.9, I43, I25.5, K76.1, I13.0-I13.2, J81** | **BNP ≥150 pg/mL (or NT-proBNP ≥600 pg/mL) at the screening visit (Visit 1) or a BNP ≥100 pg/mL (or NT-proBNP ≥400 pg/mL)**  **and a hospitalization for HF within the last 12 months** | **NT-proBNP ≥600 pg/mL**  **OR**  **NT-proBNP ≥400 pg/mL and a hospitalization for HF within the last 12 months** | **ECG: SwedeHF variable**  **NT-proBNP: SwedeHF variable**  **Prior HF hospitalization: NPR ICD codes: I50, I11.0, I42.0, I42.3-I42.9, I43, I25.5, K76.1, I13.0-I13.2, J81** |
| **Exclusion criteria** | | | | | |
| **PARAGON** | **Definition** | **Source and codes** | **PARADIGM** | **Definition** | **Source and codes** |
| ﻿1. Any prior echocardiographic measurement of EF <40% | If no prior EF measurement available, patients were considered eligible | SwedeHF | -- | -- | -- |
| 2. Acute coronary syndrome (including myocardial infarction), cardiac surgery, other major CV surgery, or urgent PCI within the 3 months prior to visit 1 or an elective PCI within 30 days prior to visit 1 | Unstable angina, STEMI and NSTEMI, PCI, CABG, aortic valve surgery, mitral valve surgery, ascending aorta surgery within 30 days prior to registration in SwedeHF | NPR:  unstable angina - I20.0  STEMI and NSTEMI MI – I21  Subsequent STEMI and NSTEMI MI – I22  PCI – procedure codes FNG  CABG - procedure codes FNA, FNB, FNC,FND,FNE,FNF,FNH.  Aortic valve surgery - procedure codes FMA00, FMA10, FMA20, FMA96, FMC00, FMC10, FMC20, FMC96, FMD00, FMD10, FMD20, FMD30, FMD33, FMD40, FMD96, FMW96  Mitral valve surgery - procedure codes FKA, FKB, FKC, FKD, FKW96, excl. FKA32 | - Acute coronary syndrome, cardiac, carotid, or other major cardiovascular surgery, PCI, within the 3 months prior to Visit 1 | As in PARAGON | As in PARAGON |
| 3. Any clinical event within the 6 months prior to visit 1 that could have reduced the EF unless an echocardiographic measurement was performed after the event confirming the EF to be ≥45% | -- | -- | -- | -- | -- |
| 4. Current acute decompensated HF requiring augmented therapy with diuretic agents, vasodilator agents, and/or inotropic drugs | -- | -- | Current acute decompensated heart failure (exacerbation of chronic heart failure manifested by signs and symptoms that may require intravenous therapy) | -- | -- |
| 5. Patients who require treatment with 2 or more of the following: an ACEi, an ARB, or a renin inhibitor | Concomitant use of ACEi and ARB at baseline | SwedeHF | Requirement for treatment with both ACEis and ARBs | Concomitant use of ACEI and ARB at baseline | SwedeHF |
| 6. History of hypersensitivity to any of the study drugs or to drugs of similar chemical classes | -- | -- | - History of hypersensitivity or allergy to any of the study drugs, drugs of similar chemical classes, ACEi, ARBs, or neprilysin inhibitors, as well as known or suspected contraindications to the study drugs.  - Previous history of intolerance to recommended target doses of ACEIs or ARBs. | -- | -- |
| **7. Patients with a known history of angioedema** | **History of angioedema** | **NPR: ICD code T78.3** | **Patients with a known history of angioedema** | **History of angioedema** | **NPR ICD code T78.3** |
| 8. Probable alternative diagnoses that in the opinion of the investigator could account for the patient’s HF symptoms (i.e., dyspnea, fatigue), such as significant pulmonary disease (including primary **pulmonary hypertension**), anemia, or obesity. Specifically, patients with the following are excluded: a) Severe pulmonary disease including COPD (i.e., requiring home oxygen, chronic nebulizer therapy, or chronic oral steroid therapy or hospitalized for pulmonary decompensation within 12 months) or  b) Hemoglobin <10 g/dl, or c) BMI >40 kg/m2 | **a) PAH (within 1 year**)  b) hemoglobin at baseline  c) BMI at baseline | **a) NPR ICD code I27.0 (within 1 year)**  b) SwedeHF  c) SwedeHF | History of severe pulmonary disease. | a) PAH (within 1 year) | a) NPR ICD code I27.0 (within 1 year) |
| 9. Patients with any of the following: a) SBP ≥180 mm Hg at visit 1, or b) SBP >150 mm Hg and <180 mm Hg at visit 1 unless the patient is receiving 3 or more antihypertensive drugs. Antihypertensive drugs include but are not limited to a thiazide or other diuretic, mineralocorticoid, ACEI, ARB, beta blocker, and calcium channel blocker, or  c) **SBP <110 mm Hg at visit 1,** or d) SBP <100 mm Hg or symptomatic hypotension as determined by the investigator at visit 103 or visit 199/201 | a) SBP at baseline  b) SBP at baseline and use of diuretics, MRA, ACEI, ARB and betablocker. No use of CCB available in SwedeHF, patients were considered as taking calcium channel blockers  **c) SBP at baseline <110 mm Hg at the baseline**  d) -- | a) SwedeHF  b) SwedeHF  **c) SwedeHF**  d) -- | Symptomatic hypotension and/or a **SBP <100 mmHg at Visit 1 (screening)** or <95 mmHg at Visit 3 or at Visit 5 (randomization) | **SBP <100 mmHg at baseline** | **SwedeHF** |
| 10. Use of other investigational drugs at the time of enrollment, or within 30 days or 5 half-lives of enrollment, whichever is longer | -- | -- | -- | -- | -- |
| 11. Patients with history of any dilated cardiomyopathy, including peripartum cardiomyopathy, chemotherapy-induced cardiomyopathy, or viral myocarditis | History of dilated cardiomyopathy | SwedeHF | Diagnosis of peripartum- or chemotherapy-induced cardiomyopathy within the 12 months prior to Visit 1 | -- | -- |
| 12. Evidence of right-sided HF in the absence of left-sided structural heart disease | -- | -- | -- | -- | -- |
| **13. Known pericardial constriction, genetic hypertrophic cardiomyopathy, or infiltrative cardiomyopathy** | **Chronic constrictive pericarditis, hypertrophic cardiomyopathy, eosinophilic cardiomyopathy, other restrictive cardiomyopathy, other/unknown cardiomyopathy** | **NPR**  **Chronic constrictive pericarditis - I31.1**  **HCM - I42.1, I42.2**  **Endomyocardial (eosynophilic) disease - I42.3**  **Other restrictive cardiomyopathies - I42.5**  **Other cardiomyopathies - I42.8, I42.9** | -- | -- | -- |
| 14. Clinically significant congenital heart disease that could be the cause of the patient’s symptoms and signs of HF | History of congenital heart disease | NPR:  Congenital malformation of cardiac chambers and connections – Q20  Congenital malformations of cardiac septa – Q21 (includes Q21.0 ventricular septal defect)  Q21.1 - Atrial septal defect  Q21.2 Atrioventricular septal defect  Q21.3 Tetralogy of Fallot  Q21.4 Aortopulmonary septal defect  Q21.8 Other congenital malformations of cardiac septa  Q21.9 Congenital malformation of cardiac septum, unspecified  Congenital malformations of pulmonary and tricuspid valves – Q22  Q22.0 - Pulmonary valve atresia  Q22.1 - Congenital pulmonary valve stenosis  Q22.2 - Congenital pulmonary valve insufficiency  Q22.3 - Other congenital malformations of pulmonary valve  Q22.4 - Congenital tricuspid stenosis  Q22.5 - Ebstein's anomaly  Q22.6 - Hypoplastic right heart syndrome  Q22.8 - Other congenital malformations of tricuspid valve  Q22.9 - Congenital malformation of tricuspid valve, unspecified  Congenital malformations of aortic and mitral valves – Q23  Q23.0 - Congenital stenosis of aortic valve  Q23.1 - Congenital insufficiency of aortic valve  Q23.2 - Congenital mitral stenosis  Q23.3 - Congenital mitral insufficiency  Q23.4 - Hypoplastic left heart syndrome  Q23.8 - Other congenital malformations of aortic and mitral valves  Q23.9 - Congenital malformation of aortic and mitral valves, unspecified  Other congenital malformations of heart – Q24  Q24.0 - Dextrocardia  Q24.1 - Levocardia  Q24.2 - Cor triatriatum  Q24.3 - Pulmonary infundibular stenosis  Q24.4 - Congenital subaortic stenosis  Q24.5 - Malformation of coronary vessels  Q24.6 - Congenital heart block  Q24.8 - Other specified congenital malformations of heart  Q24.9 - Congenital malformation of heart, unspecified  Congenital malformations of great arteries – Q25  Q25 - Congenital malformations of great arteries  Q25.0 - Patent ductus arteriosus  Q25.1 - Coarctation of aorta  Q25.2 - Atresia of aorta  Q25.3 - Supravalvular aortic stenosis  Q25.4 - Other congenital malformations of aorta  Q25.5 - Atresia of pulmonary artery  Q25.6 - Stenosis of pulmonary artery  Q25.7 - Other congenital malformations of pulmonary artery  Q25.8 - Other congenital malformations of other great arteries  Q25.9 - Congenital malformation of great arteries, unspecified  Congenital malformations of great veins – Q26  Q26.0 - Congenital stenosis of vena cava  Q26.1 - Persistent left superior vena cava  Q26.2 - Total anomalous pulmonary venous connection  Q26.3 - Partial anomalous pulmonary venous connection  Q26.4 - Anomalous pulmonary venous connection, unspecified  Q26.5 - Anomalous portal venous connection  Q26.6 - Portal vein-hepatic artery fistula  Q26.8 - Other congenital malformations of great veins  Q26.9 - Congenital malformation of great vein, unspecified  Other congenital malformations of peripheral vascular system – Q27  Q27.0 - Congenital absence and hypoplasia of umbilical artery  Q27.1 - Congenital renal artery stenosis  Q27.2 - Other congenital malformations of renal artery  Q27.3 - Arteriovenous malformation (peripheral)  Q27.4 - Congenital phlebectasia  Q27.8 - Other specified congenital malformations of peripheral vascular system  Q27.9 - Congenital malformation of peripheral vascular system, unspecified  Other congenital malformations of circulatory system – Q28  Q28.0 - Arteriovenous malformation of precerebral vessels  Q28.1 - Other malformations of precerebral vessels  Q28.2 - Arteriovenous malformation of cerebral vessels  Q28.3 - Other malformations of cerebral vessels  Q28.8 - Other specified congenital malformations of circulatory system  Q28.9 - Congenital malformation of circulatory system, unspecified | -- | -- | -- |
| 15. Presence of hemodynamically significant valvular heart disease in the opinion of the investigator | -- | -- | - Presence of haemodynamically significant mitral and/or aortic valve disease, except mitral regurgitation secondary to left ventricular dilatation.  - Presence of other haemodynamically significant obstructive lesions of the left ventricular outflow tract, including aortic and subaortic stenosis. | -- | -- |
| 16. Stroke, transient ischemic attack, carotid surgery, or carotid angioplasty within the 3 months prior to visit 1 | Stroke or TIA within 3 months prior to baseline | NPR:  I60- Nontraumatic subarachnoid hemorrhage  I61- Nontraumatic intracerebral hemorrhage  I62- Other and unspecified nontraumatic intracranial hemorrhage  I63- Cerebral infarction  I64- Acute cerebrovascular disease without specification whether it is bleeding or infarct | Stroke, transient ischemic attack, carotid surgery, or carotid angioplasty within the 3 months prior to visit 1 | Stroke or TIA within 3 months prior to baseline | As in PARAGON |
| 17. Coronary or carotid artery disease or valvular heart disease likely to require surgical or percutaneous intervention during the trial | -- | -- | Coronary or carotid artery disease likely to require surgical or percutaneous intervention within the 6 months after Visit 1 | -- | -- |
| 18. Life-threatening or uncontrolled dysrhythmia, including symptomatic or sustained ventricular tachycardia and AF or atrial flutter with a resting ventricular rate >110 beats per minute | ECG showing AF and HR above 110 bpm at baseline | ECG: SwedeHF  HR: SwedeHF | Documented untreated ventricular arrhythmia with syncopal episodes within the 3 months prior to Visit 1.  Symptomatic bradycardia or second- or third-degree atrioventricular block without a pacemaker. | -- | -- |
| 19. Patients with a CRT | CRT use at baseline | SwedeHF | Implantation of a CRT within 3 months prior Visit 1 or intent to implant a CRT. | CRT use at baseline | SwedeHF |
| 20. Patients with prior major organ transplant or intent to transplant (i.e., on transplant list) | History of renal / heart / lung / liver / bone / bone marrow / intestine / pancreas transplantation | NPR:  Kidney transplant status – Z94.0, procedure codes KAS  Heart transplant status - Z94.1, procedure codes FQA  Lung transplant status - Z94.2, procedure codes GDG  Heart and lungs transplant status - Z94.3, procedure codes FQB  Liver transplant status - Z94.4  Bone transplant status - Z94.6  Other transplants – Z94.8 | History of heart transplant or on a transplant list or with left ventricular assistance device | History of heart transplant | NPR:  Heart transplant status - Z94.1, procedure codes FQA  Heart and lungs transplant status - Z94.3, procedure codes FQB |
| 21. Any surgical or medical condition that in the opinion of the investigator may place the patient at higher risk from his/her participation in the study or is likely to prevent the patient from complying with the requirements of the study or completing the study | History of dementia/organic amnesic syndrome / delirium / other mental disorder or personality and behavioral disorders due to brain disease / unspecified mental disorder/mental disorders due to psychoactive substances / schizophrenia / manic episode / bipolar affective disorder. | NPR:  Mental disorders due to known physiological conditions  **F01 - Vascular dementia**  **F02 - Dementia in other diseases classified elsewhere**  **F03 - Unspecified dementia**  F04 - Amnestic disorder due to known physiological condition  F05 - Delirium due to known physiological condition  F06 - Other mental disorders due to known physiological condition  F07 - Personality and behavioral disorders due to known physiological condition  F09 - Unspecified mental disorder due to known physiological condition  Mental and behavioral disorders due to psychoactive substance use  F10 - Alcohol related disorders  F11 - Opioid related disorders  F12 - Cannabis related disorders  F13 - Sedative, hypnotic, or anxiolytic related disorders  F14 - Cocaine related disorders  F15 - Other stimulant related disorders  F16 - Hallucinogen related disorders  F18 - Inhalant related disorders  F19 - Other psychoactive substance related disorders  Schizophrenia, schizotypal, delusional, and other non-mood psychotic disorders  F20 - Schizophrenia  F21 - Schizotypal disorder  F22 - Delusional disorders  F23 - Brief psychotic disorder  F24 - Shared psychotic disorder  F25 - Schizoaffective disorders  F28 - Other psychotic disorder not due to a substance or known physiological condition  F29 - Unspecified psychosis not due to a substance or known physiological condition  Mood [affective] disorders  F30 - Manic episode  F31 - Bipolar disorder  ICD codes within 1 year from the SwedeHF registration | -- | -- | -- |
| 22. Any surgical or medical condition that might significantly alter the absorption, distribution, metabolism, or excretion of study drugs, including but not limited to any of the following: any history of pancreatic injury, pancreatitis, or evidence of impaired pancreatic function/injury within the past 5 years | History of pancreatic diseases within 5 years | NPR:  K86.0, K86.1 - Pacreatitis | Any surgical or medical condition which might significantly alter the absorption, distribution, metabolism, or excretion of study drugs, including, but not  limited to, any of the following:  - History of active inflammatory bowel disease during the 12 months before Visit 1.  - Active duodenal or gastric ulcers during the 3 months prior to Visit 1.  - Evidence of hepatic disease as determined by any one of the following: aspartate aminotransferase or alanine aminotransferase values exceeding 2×  upper limit of normal at Visit 1, history of hepatic encephalopathy, history of oesophageal varices, or history of porto-caval shunt.  - Current treatment with cholestyramine or colestipol resins. | Liver disease within 1 year.  Duodenal or gastric ulcers within 3 months | Pacreatitis - K86.0, K86.1  Gastric and duodenal ulcers: K25.0-25.3; K26.0-26.3  Liver disease - K70-77, B18 |
| 23. Evidence of hepatic disease as determined by any 1 of the following: SGOT (AST) or SGPT (ALT) values exceeding 3? the upper limit of normal, bilirubin >1.5 mg/dl at visit 1 | History of liver disease | NPR:  K70 - Alcoholic liver disease  K71 - Toxic liver disease  K72 - Hepatic failure, not elsewhere classified  K73 - Chronic hepatitis, not elsewhere classified  K74 - Fibrosis and cirrhosis of liver  K75 - Other inflammatory liver diseases  K76 - Other diseases of liver  K77 - Liver disorders in diseases classified elsewhere  B18 - Chronic viral hepatitis  ICD codes within 1 year from the SwedeHF registration | -- | -- | -- |
| **24. Patients with 1 of the following: a) eGFR <30 ml/min/1.73 m2 as calculated by the Modification in Diet in Renal Disease (MDRD) formula at visit 1, or b) eGFR <25 ml/min/1.73 m2 at visit 103 or visit 199/201, or c) eGFR reduction >35% (compared with visit 1) at visit 103 or visit 199/201** | **eGFR <30 ml/min/1.73 m2** | **SwedeHF** | **eGFR <30 mL/min/1.73 m2 at Visit 1 (screening), Visit 3 (end of enalapril run-in), or Visit 5 (end of LCZ696 run-in**  **and randomization) or >35% decline in eGFR between Visit 1 and Visit 3 or between Visit 1 and Visit 5.** | **eGFR <30 ml/min/1.73 m2** | **SwedeHF** |
| 25. Presence of known functionally significant bilateral renal artery stenosis | -- | -- | -- | -- | -- |
| 26. Patients with either of the following: a) Serum potassium >5.2 mmol/l (mEq/l) at visit 1 b) Serum potassium >5.4 mmol/l (mEq/l) at visit 103 or visit 199/201 | Serum potassium >5.2 mmol/l (mEq/l) | SwedeHF | Serum potassium >5.2 mmol/L at Visit 1 (screening) or >5.4 mmol/L at Visit 3 or Visit 5 (randomization). | Serum potassium >5.2 mmol/l (mEq/l) | SwedeHF |
| 27. History or presence of any other disease with a life expectancy of <3years | -- | -- | Presence of any other disease with a life expectancy of <5 years. | -- | -- |
| 28. History of noncompliance to medical regimens and patients who are considered potentially unreliable | -- | -- | -- | -- | -- |
| 29. History or evidence of drug or alcohol abuse within the past 12 months | History of drug/alcohol abuse within 12 months | NPR:  F10, E52, G621, I426, K292, K700, K703, K709, T51, Z502, Z714, F11, F12, F13, F14, F15, F16, F18, F19 | -- | -- | -- |
| 30. Persons directly involved in the execution of this protocol | -- | -- | -- | -- | -- |
| 31. History of malignancy of any organ system (other than localized basal or squamous cell carcinoma of the skin or localized prostate cancer), treated or untreated, within the past 5 years, regardless of whether there is evidence of local recurrence or metastases | History of malignancies other than localized basal/squamous cell carcinoma 5 years to baseline | NPR:  All malignancies excl:  C44.0B, C44.0C, C44.0D, C44.0E, C44.0S - Basal/ squamous cell carcinoma of skin of lip  C44.1B, C44.1C, C44.1D, C44.1E, C44.1S - Basal/ squamous cell carcinoma of skin of eyelid, including canthus  C44.2B, C44.2C, C44.2 D, C44.2E, C44.2S - Basal/ squamous cell carcinoma of skin of ear and external auricular canal  C44.3B, C44.3C, C44.3D, C44.3E, C44.3S - Basal/ squamous cell carcinoma of skin of other and unspecified parts of face  C44.4B, C44.4C, C44.4D, C44.4E, C44.4S - Basal/ Squamous cell carcinoma of skin of scalp and neck  C44.5B, C44.5C, C44.5D, C44.5E, C44.5S - Squamous cell carcinoma of skin of trunk  C44.6B, C44.6C, C44.6D, C44.6E, C44.6S - Basal/ Squamous cell carcinoma of skin of upper limb, including shoulder  C44.7B, C44.7C, C44.7D, C44.7E, C44.7S - Basal/ Squamous cell carcinoma of skin of lower limb, including hip  C44.8B, C44.8C, C44.8D, C44.8E, C44.8S - Basal/ Squamous cell carcinoma of overlapping sites of skin  C44.9B, C44.9C, C44.9D, C44.9E, C44.9S - Basal/ Squamous cell carcinoma of skin, unspecified  (Swedish ICD-10 codes version) | -- | -- | -- |
| 32. Pregnant or nursing (lactating) women, where pregnancy is defined as the state of a female after conception and until the termination of gestation, confirmed by a positive human chorionic gonadotropin laboratory test | -- | -- | -- | -- | -- |
| 33. Women of child-bearing potential, defined as all women physiologically capable of becoming pregnant, unless they are using highly effective methods of contraception during dosing and for 7 days off study drug ACEi | -- | -- | -- | -- | -- |

Abbreviations – SwedeHF: Swedish heart failure registry; NPR: national patient registry; EF: ejection fraction; HF: heart failyre; NYHA: New York heart association; ACEi: angiotensin converting enzyme inhibitor; ARB: angiotensin receptor blocker; BNP: B-type natriuretic petide; NT-proBNP: N-terminal pro-B- type natriuretic peptide; CV: cardiovascular: PCI: percutaneous coronary intervention; CABG: coronary artery by-pass graft; MI: myocardial infarction; STEMI: ST segment elevation myocardial infarction; NSTEMI: Non-ST segment elevation myocardial infarction; BMI: body mass index; PAH: pulmonary arterial hypertension; SBP: systolic blood pressure; HCM: hypertrophic cardiomyopathy; HR: heart rate; CRT: cardiac resynchronization therapy; SGOT: serum glutamic oxaloacetic transaminase; aspartate animotransferase; SGPT: serum glutamic pyruvic transaminase; ALT: alanine transaminase; eGFR: estimated glomerular filtration rate; ECG: electrocardiogram; AF atrial fibrillation; LA: left atria; LVH: left ventricular hypertropy

**Supplementary Table 2. Baseline characteristics according to the ejection fraction group**

|  | **EF≥50%** | **EF=40-49%** | **EF<40%** |
| --- | --- | --- | --- |
| **n** | **7172 (19%)** | **9134 (24%)** | **21484 (57%)** |
| **Demographic/Organizational/Socioeconomic Characteristics** | | | |
| **Sex = Males** | 3663 ( 51.1) | 5980 ( 65.5) | 15775 ( 73.4) |
| **Age, median [IQR]** | 77.0 [69.0, 83.0] | 74.0 [66.0, 81.0] | 72.0 [64.0, 80.0] |
| **Follow-up referral = Specialty** | 3534 ( 50.7) | 5608 ( 63.1) | 16132 ( 77.5) |
| **Follow-up referral HF nurse-led clinic** | 3386 ( 49.3) | 4707 ( 53.3) | 11760 ( 57.0) |
| **Year of registration** |  |  |  |
| **2000-2005** | 291 ( 4.1) | 413 ( 4.5) | 1110 ( 5.2) |
| **2006-2011** | 2933 ( 40.9) | 3473 ( 38.0) | 8569 ( 39.9) |
| **2012-2016** | 3948 ( 55.0) | 5248 ( 57.5) | 11805 ( 54.9) |
| **Marital status** |  |  |  |
| **Married** | 3385 ( 47.2) | 4743 ( 52.0) | 11037 ( 51.5) |
| **Single** | 1899 ( 26.5) | 2682 ( 29.4) | 7160 ( 33.4) |
| **Widowed** | 1884 ( 26.3) | 1700 ( 18.6) | 3246 ( 15.1) |
| **Educational level** |  |  |  |
| **Compulsory school** | 3304 ( 47.0) | 3830 ( 42.6) | 9141 ( 43.3) |
| **Secondary school** | 2563 ( 36.5) | 3588 ( 39.9) | 8520 ( 40.4) |
| **University** | 1162 ( 16.5) | 1578 ( 17.5) | 3436 ( 16.3) |
| **Income** |  |  |  |
| **Low** | 2686 ( 37.5) | 2885 ( 31.6) | 6866 ( 32.0) |
| **Medium** | 2403 ( 33.5) | 3057 ( 33.5) | 6998 ( 32.6) |
| **High** | 2079 ( 29.0) | 3183 ( 34.9) | 7579 ( 35.3) |
| **Clinical Characteristics** | | | |
| **HF duration ≤ 6 months** | 4555 ( 64.8) | 5369 ( 59.8) | 11979 ( 56.6) |
| **Previous HF hospitalization within 9 months** | 2029 ( 28.3) | 2339 ( 25.6) | 9154 ( 42.6) |
| **Previous HF hospitalization within 12 months** | 2152 ( 30.0) | 2505 ( 27.4) | 9626 ( 44.8) |
| **NYHA class** |  |  |  |
| **I** | 978 ( 16.5) | 1328 ( 16.3) | 2013 ( 10.3) |
| **II** | 2910 ( 49.1) | 4478 ( 54.8) | 9737 ( 49.6) |
| **III** | 1937 ( 32.7) | 2263 ( 27.7) | 7472 ( 38.1) |
| **IV** | 107 ( 1.8) | 101 ( 1.2) | 398 ( 2.0) |
| **ECG** |  |  |  |
| **Atrial fibrillation** | 2820 ( 40.5) | 3099 ( 34.6) | 6510 ( 30.8) |
| **PM/other rhythm** | 591 ( 8.5) | 921 ( 10.3) | 2654 ( 12.5) |
| **Sinus rhythm** | 3553 ( 51.0) | 4945 ( 55.2) | 11991 ( 56.7) |
| **Systolic blood pressure** | 130.0 [115.0, 140.0] | 130.0 [115.0, 140.0] | 120.0 [110.0, 138.0] |
| **Diastolic blood pressure** | 70.0 [65.0, 80.0] | 72.0 [65.0, 80.0] | 70.0 [65.0, 80.0] |
| **Heart rate** | 70.0 [61.0, 79.0] | 69.0 [60.0, 78.0] | 70.0 [60.0, 80.0] |
| **eGFR** |  |  |  |
| **<30 ml/min/1.73m2** | 474 ( 6.8) | 458 ( 5.1) | 1125 ( 5.3) |
| **30-<60 ml/min/1.73m2** | 3161 ( 45.3) | 3464 ( 38.7) | 8403 ( 39.7) |
| **≥60 ml/min/1.73m2** | 3339 ( 47.9) | 5036 ( 56.2) | 11620 ( 54.9) |
| **Haemoglobin (g/L), median [IQR]** | 131.0 [120.0, 142.0] | 135.0 [123.0, 146.0] | 136.0 [125.0, 147.0] |
| **NT-proBNP (pg/ml), median [IQR]** | 1378.0 [570.0, 2940.0] | 1331.0 [556.2, 2880.0] | 2242.0 [975.0, 4900.0] |
| **Potassium (mEq/L), median [IQR]** | 4.2 [4.0, 4.5] | 4.2 [4.0, 4.5] | 4.3 [4.0, 4.6] |
| **BMI (kg/m2)** |  |  |  |
| **≤30** | 2700 ( 67.3) | 3830 ( 71.4) | 9651 ( 74.6) |
| **30-40** | 1125 ( 28.0) | 1389 ( 25.9) | 3011 ( 23.3) |
| **>40** | 186 ( 4.6) | 145 ( 2.7) | 276 ( 2.1) |
| **Treatments** | | | |
| **ACEi/ARB** | 5812 ( 81.6) | 8137 ( 89.7) | 19930 ( 93.5) |
| **MRA** | 2055 ( 28.8) | 2433 ( 26.8) | 8355 ( 39.1) |
| **Diuretic** | 5382 ( 75.4) | 5933 ( 65.2) | 15930 ( 74.4) |
| **Nitrate** | 931 ( 13.0) | 1054 ( 11.6) | 2505 ( 11.7) |
| **Antiplatelet** | 2506 ( 35.6) | 3914 ( 43.4) | 9416 ( 44.4) |
| **Anticoagulant** | 3452 ( 48.3) | 4170 ( 45.8) | 9945 ( 46.4) |
| **Statin** | 3160 ( 44.2) | 4796 ( 52.7) | 11269 ( 52.6) |
| **Beta-blocker** | 5881 ( 82.2) | 8035 ( 88.2) | 19971 ( 93.1) |
| **Digoxin** | 998 ( 13.9) | 1150 ( 12.6) | 3137 ( 14.7) |
| **HF device** |  |  |  |
| **CRT-P** | 35 ( 0.5) | 117 ( 1.3) | 630 ( 3.0) |
| **CRT-D** | 38 ( 0.6) | 101 ( 1.1) | 908 ( 4.3) |
| **ICD** | 68 ( 1.0) | 147 ( 1.7) | 909 ( 4.3) |
|  | | | |
| **Anemia** | 2175 ( 31.9) | 2435 ( 28.1) | 5692 ( 27.7) |
| **Smoking** |  |  |  |
| **Current** | 421 ( 8.2) | 750 ( 11.2) | 2178 ( 13.0) |
| **Former** | 2119 ( 41.5) | 2969 ( 44.2) | 7944 ( 47.2) |
| **Never** | 2569 ( 50.3) | 2993 ( 44.6) | 6692 ( 39.8) |
| **Hypertension** | 4486 ( 63.7) | 4845 ( 54.1) | 10166 ( 48.6) |
| **Diabetes** | 1700 ( 23.9) | 2014 ( 22.1) | 5127 ( 24.0) |
| **Ischemic heart disease** | 3060 ( 42.7) | 4815 ( 52.7) | 11487 ( 53.5) |
| **Myocardial infarction** | 1401 ( 19.5) | 2715 ( 29.7) | 6467 ( 30.1) |
| **Coronary revascularization** | 1614 ( 22.5) | 2947 ( 32.3) | 7048 ( 32.8) |
| **Peripheral vascular disease** | 683 ( 9.5) | 912 ( 10.0) | 2172 ( 10.1) |
| **Stroke/TIA** | 1000 ( 13.9) | 1212 ( 13.3) | 2816 ( 13.1) |
| **Atrial fibrillation** | 4005 ( 56.3) | 4546 ( 50.1) | 9755 ( 45.6) |
| **Valvular disease** | 1970 ( 27.5) | 1960 ( 21.5) | 3752 ( 17.5) |
| **COPD** | 1069 ( 14.9) | 1192 ( 13.1) | 2818 ( 13.1) |
| **Liver disease (within 1 year)** | 94 ( 1.3) | 107 ( 1.2) | 292 ( 1.4) |
| **Malignancy (within 5 years)** | 1104 ( 15.4) | 1372 ( 15.0) | 2926 ( 13.6) |
| **Pancreatic disease (within 1 year)** | 100 ( 1.4) | 111 ( 1.2) | 311 ( 1.4) |
| **Mental disease (within 1 year)** | 195 ( 2.7) | 247 ( 2.7) | 811 ( 3.8) |

Abbreviations – HF: heart failure; HFpEF: HF with preserved ejection fraction; HFmrEF: HF with mid-range ejection fraction; HFrEF: HF with reduced ejection fraction; NYHA: New York heart association; NT-proBNP: N-terminal pro-B-type natriuretic peptide; ACEi: Angiotensin converting enzyme inhibitor; ARB: angiotensin receptor blocker; MRA: mineralocorticoid receptor antagonist; ICD: implantable cardioverter defibrillator; CRT-D: cardiac resynchronization therapy-defibrillator; CRT-P: cardiac resynchronization therapy-pacemaker; COPD: chronic obstructive pulmonary disease; IQR: interquartile range; TIA: Transient Ischemic Attack; ECG: electrocardiogram

**Supplementary Table 3. Eligibility for sacubitril/valsartan based on PARADIGM-HF/PARAGON-HF selection criteria in the main study population (missing data imputed) by ejection fraction phenotype.**

|  |  | **EF≥50%** | **EF=40-49%** | **EF<40%** |
| --- | --- | --- | --- | --- |
|  | **No of patients** | **7172 (19%)** | **9134 (24%)** | **21484 (57%)** |
|  | **Inclusion criteria** |  |  |  |
| 1 | Written informed consent must be obtained before any assessment is performed | Assumed 100% | Assumed 100% | Assumed 100% |
| **2** | **Age ≥50 in PARAGON-HF / ≥18 in PARADIGM-HF** | **6902 (96.2%)** | **8652 (94.7%)** | **21484 (100%)** |
| 3 | LVEF | 100% | 100% | 100% |
| 4 | Diuretic treatment at discharge for PARAGON-HF /ACEi or ARB equivalent to enalapril 10 mg/day and BBl at discharge for PARADIGM-HF | 5403 (75.3%) | 5955 (65.2%) | 15428 (71.8%) |
| **5** | **NYHA II-IV** | **5989 (83.5%)** | **7651 (83.8%)** | **19270 (89.7%)** |
| 6 | Structural heart disease for PARAGON-HF | Assumed 100% | Assumed 100% | -- |
| **7** | **NT-proBNP criteria** | **5779 (80.6%)** | **7326 (80.2%)** | **18623 (86.7%)** |
|  | Eligible (literal scenario) only inclusion criteria | 4194 (58.5%) | 4725 (51.7%) | 11927 (55.5%) |
|  | Eligible (pragmatic scenario) only inclusion criteria | 4986 (69.5%) | 6206 (67.9%) | 17036 (79.3%) |
|  | **Exclusion criteria** |  |  |  |
| 1 | Any prior echocardiographic measurement of LVEF <40% for PARAGON-HF | 6543 (91.2%) | 7897 (86.5%) | 21484 (100%) |
| 2 | Acute coronary syndrome/major cardiovascular interventions | 6781 (94.5%) | 8210 (89.9%) | 19166 (89.2%) |
| 3 | Any clinical event within the 6 months prior to visit 1 that could have reduced the LVEF (PARAGON-HF) | Assumed 100% | Assumed 100% | -- |
| 4 | Current acute decompensated HF | 100% | 100% | 100% |
| 5 | Patients with both ACEi and ARB at discharge | 7071 (98.6%) | 9003 (98.6%) | 20955 (97.5%) |
| 6 | History of hypersensitivity to any of the study drugs | Assumed 100% | Assumed 100% | Assumed 100% |
| **7** | **Known history of angioedema** | **7172 (100%)** | **9132 (100%)** | **21484 (100%)** |
| **8** | **Pulmonary arterial hypertension** | **7111 (99.1%)** | **9096 (99.6%)** | **21440 (99.8%)** |
| 8 | BMI >40 kg/m2 | 6861 (95.7%) | 8899 (97.4%) | 21484 (100%) |
| 8 | Hemoglobin <10 g/dL | 6999 (97.6%) | 8968 (98.2%) | 21484 (100%) |
| 9 | SBP ≥ 180 mmHg (PARAGON-HF) | 7021 (97.9%) | 8975 (98.3%) | 21484 (100%) |
| 9 | SBP >150 mmHg and <180 mmHg at baseline, unless the patient is receiving 3 or more antihypertensive drugs (assuming all receive CCBs) | 7083 (98.8%) | 9070 (99.3%) | 21484 (100%) |
| **9** | **SBP <110 mmHg (PARAGON-HF) / <100 mmHg (PARADIGM-HF)** | **6277 (87.5%)** | **7847 (85.9%)** | **19654 (91.5%)** |
| 9 | SBP <100 mm Hg or symptomatic hypotension as determined by the investigator at visit 103 or visit 199/201 | Assumed 100% | Assumed 100% | Assumed 100% |
| 10 | Use of other investigational drugs | Assumed 100% | Assumed 100% | Assumed 100% |
| 11 | History of dilated cardiomyopathy | 6768 (94.4%) | 8349 (91.4%) | 21484 (100%) |
| 12 | Evidence of right-sided HF in the absence of left-sided structural heart disease | Assumed 100% | Assumed 100% | -- |
| **13** | **Pericardial constriction/hypertrophic or infiltrative cardiomyopathy (PARAGON-HF)** | **6748 (94.1%)** | **8744 (95.7%)** | **--** |
| 14 | Clinically significant congenital heart disease | 7066 (98.5%) | 9024 (98.8%) | 21484 (100%) |
| 15 | Hemodynamically significant valvular disease | Assumed 100% | Assumed 100% | Assumed 100% |
| 16 | Stroke or TIA within 3 months prior to index date | 7113 (99.2%) | 9047 (99%) | 21171 (98.5%) |
| 17 | Coronary or carotid or valvular heart disease requiring intervention | Assumed 100% | Assumed 100% | Assumed 100% |
| 18 | AF and HR >110 | 7096 (98.9%) | 9043 (99%) | 21484 (100%) |
| 19 | CRT | 7094 (98.9%) | 8912 (97.6%) | 19939 (92.8%) |
| 20 | Previous major transplant | 7130 (99.4%) | 9090 (99.5%) | 21466 (99.9%) |
| **21** | **Trial scenario - Mental disorders (PARAGON-HF)** | **6977 (97.3%)** | **8887 (97.3%)** | **--** |
| 21 | Pragmatic scenario - Mental disorders (PARAGON-HF) | 7134 (99.5%) | 9090 (99.5%) | -- |
| 22 | Pancreatic disease within 5 years prior to index date (PARAGON-HF) / Liver disease within 1 year or Crohn within 1 year or Duodenal or gastric ulcers within 3 months (PARADIGM-HF) | 7160 (99.8%) | 9123 (99.9%) | 21130 (98.4%) |
| 23 | History of liver disease (PARAGON-HF) | 7078 (98.7%) | 9027 (98.8%) | -- |
| **24** | **eGFR < 30 ml/min/1.73m2** | **6681 (93.2%)** | **8666 (94.9%)** | **20343 (94.7%)** |
| 24 | eGFR <25 ml/min/1.73 m2 at visit 103 or visit 199/201 | Assumed 100% | Assumed 100% | Assumed 100% |
| 24 | eGFR reduction >35% (compared with visit 1) at visit 103 or visit 199/201 | Assumed 100% | Assumed 100% | Assumed 100% |
| 25 | Presence of known functionally significant bilateral renal artery stenosis | Assumed 100% | Assumed 100% | Assumed 100% |
| 26 | Potassium > 5.2 mmol/l | 7052 (98.3%) | 8991 (98.4%) | 21117 (98.3%) |
| 26 | Serum potassium >5.4 mmol/l at visit 103 or visit 199/201 | Assumed 100% | Assumed 100% | Assumed 100% |
| 27 | Life expentance < 3 (PARAGON-HF) /5 (PARADIGM-HF) years | Assumed 100% | Assumed 100% | Assumed 100% |
| 28 | Noncompliance | Assumed 100% | Assumed 100% | Assumed 100% |
| 29 | Drug or alcohol abuse within the past 12 months | 7129 (99.4%) | 9093 (99.6%) | 21484 (100%) |
| 30 | Persons directly involved in the execution of this protocol | Assumed 100% | Assumed 100% | Assumed 100% |
| 31 | Malignancy within the past 5 years prior to index (PARAGON-HF) | 6068 (84.6%) | 7762 (85%) | -- |
| 32 | Pregnant or nursing (lactating) women | Assumed 100% | Assumed 100% | Assumed 100% |
| 33 | Women of child-bearing potential | Assumed 100% | Assumed 100% | Assumed 100% |
|  | Eligible (literal scenario) only exclusion criteria | 3125 (43.6%) | 3608 (39.5%) | 14388 (67%) |
|  | Eligible (pragmatic scenario) only exclusion criteria | 5465 (76.2%) | 7084 (77.6%) | 18661 (86.9%) |
|  | Eligible (literal scenario) | 1804 (25.2%) | 1804 (19.8%) | 8061 (37.5%) |
|  | Eligible (pragmatic scenario) | 3710 (51.7%) | 4720 (51.7%) | 14474 (67.4%) |

Eligibility estimates reported as numbers and percentages and representing the remaining cohort after applying the respective inclusion/exclusion criteria. The criteria were not ordered and mutually exclusive.

Variables included in the pragmatic scenario in **bold**.

Abbreviations – HF: heart failure; HFpEF: heart failure with preserved ejection fraction: EF: ejection fraction; NYHA: New York Heart Association; NT-proBNP: N-terminal pro-B-type natriuretic peptide; ACEi: Angiotensin converting enzyme inhibitor; ARB: angiotensin receptor blocker; CRT: cardiac resynchronization therapy; SBP: systolic blood pressure; BMI: body mass index; AF: atrial fibrillation; HR: heart rate; PARAGON-HF The Prospective Comparison of ARNI [angiotensin receptor neprilysin inhibitor] with ARB [angiotensin- receptor blockers] Global Outcomes in HF with Preserved Ejection Fraction trial; PARADIGM-HF: Prospective Comparison of ARNI With ACEI to Determine Impact on Global Mortality and Morbidity in Heart Failure; eGFR: estimated glomerular filtration rate; TIA:Transient Ischemic Attack; ECG: electrocardiogram

**Supplementary Table 4. Eligibility for sacubitril/valsartan based on PARADIGM-HF/PARAGON-HF selection criteria according to the ejection fraction group: consistency analysis**

|  |  | **EF<50%** | | **EF≥40%** | | **EF≥50%** | | **EF=40-49%** | | **EF<40%** | |
| --- | --- | --- | --- | --- | --- | --- | --- | --- | --- | --- | --- |
|  | **Criteria** | **Complete case** | **Missing as eligible** | **Complete case** | **Missing as eligible** | **Complete case** | **Missing as eligible** | **Complete case** | **Missing as eligible** | **Complete case** | **Missing as eligible** |
|  | **No of patients** | **11949** | **30618** | **4368** | **16306** | **1859** | **7172** | **2509** | **9134** | **9440** | **21484** |
| **Inclusion criteria** | | | | | | | | | | | |
| 1 | Written informed consent must be obtained before any assessment is performed | Assumed 100% | Assumed 100% | Assumed 100% | Assumed 100% | Assumed 100% | Assumed 100% | Assumed 100% | Assumed 100% | Assumed 100% | Assumed 100% |
| **2** | **Age ≥50 in PARAGON-HF / ≥18 in PARADIGM-HF** | **11817 (98.9%)** | **30136 (98.4%)** | **4168 (95.4%)** | **15554 (95.4%)** | **1791 (96.3%)** | **6902 (96.2%)** | **2377 (94.7%)** | **8652 (94.7%)** | **9440 (100%)** | **21484 (100%)** |
| 3 | LVEF | 100% | 100% | 100% | 100% | 100% | 100% | 100% | 100% | 100% | 100% |
| 4 | Diuretic treatment at discharge for PARAGON-HF /ACEi or ARB equivalent to enalapril 10 mg/day and BBl at discharge for PARADIGM-HF | 8397 (70.3%) | 21244 (69.8%) | 3195 (73.1%) | 11383 (69.8%) | 1475 (79.3%) | 5412 (75.5%) | 1720 (68.6%) | 5971 (65.4%) | 6677 (70.7%) | 15273 (71.7%) |
| **5** | **NYHA II-IV** | **10595 (88.7%)** | **27277 (89.1%)** | **3750 (85.9%)** | **14000 (85.9%)** | **1606 (86.4%)** | **6194 (86.4%)** | **2144 (85.5%)** | **7806 (85.5%)** | **8451 (89.5%)** | **19471 (90.6%)** |
| 6 | Structural heart disease for PARAGON-HF | Assumed 100% | Assumed 100% | Assumed 100% | Assumed 100% | Assumed 100% | Assumed 100% | Assumed 100% | Assumed 100% | -- | -- |
| **7** | **NT-proBNP criteria** | **10222 (85.5%)** | **28220 (92.2%)** | **3606 (82.6%)** | **14711 (90.2%)** | **1547 (83.2%)** | **6464 (90.1%)** | **2059 (82.1%)** | **8247 (90.3%)** | **8163 (86.5%)** | **19973 (93%)** |
|  | Eligible (literal scenario) only inclusion criteria | 6474 (54.2%) | 17783 (58.4%) | 2561 (58.6%) | 9610 (58.9%) | 1192 (64.1%) | 4555 (63.5%) | 1369 (54.6%) | 5055 (55.3%) | 5105 (54.1%) | 12728 (59.7%) |
|  | Eligible (pragmatic scenario) only inclusion criteria | 9190 (76.9%) | 25140 (82.1%) | 3130 (71.7%) | 12475 (76.5%) | 1376 (74%) | 5573 (77.7%) | 1754 (69.9%) | 6902 (75.6%) | 7436 (78.8%) | 18238 (84.9%) |
| **Exclusion criteria** | | | | | | | | | | | |
| 1 | Any prior echocardiographic measurement of LVEF <40% for PARAGON-HF | 11488 (96.1%) | 29381 (96%) | 3701 (84.7%) | 14440 (88.6%) | 1653 (88.9%) | 6543 (91.2%) | 2048 (81.6%) | 7897 (86.5%) | -- | -- |
| 2 | Acute coronary syndrome/major cardiovascular interventions | 10779 (90.2%) | 27376 (89.4%) | 4027 (92.2%) | 14991 (91.9%) | 1761 (94.7%) | 6781 (94.5%) | 2266 (90.3%) | 8210 (89.9%) | 8513 (90.2%) | 19166 (89.2%) |
| 3 | Any clinical event within the 6 months prior to visit 1 that could have reduced the LVEF (PARAGON-HF) | Assumed 100% | Assumed 100% | Assumed 100% | Assumed 100% | Assumed 100% | Assumed 100% | Assumed 100% | Assumed 100% | -- | -- |
| 4 | Current acute decompensated HF | 100% | 100% | 100% | 100% | 100% | 100% | 100% | 100% | 100% | 100% |
| 5 | Patients with both ACEi and ARB at discharge | 11766 (98.5%) | 29966 (97.9%) | 4310 (98.7%) | 16078 (98.6%) | 1835 (98.7%) | 7073 (98.6%) | 2475 (98.6%) | 9005 (98.6%) | 9291 (98.4%) | 20961 (97.6%) |
| 6 | History of hypersensitivity to any of the study drugs | Assumed 100% | Assumed 100% | Assumed 100% | Assumed 100% | Assumed 100% | Assumed 100% | Assumed 100% | Assumed 100% | Assumed 100% | Assumed 100% |
| **7** | **Known history of angioedema** | **11949 (100%)** | **30616 (100%)** | **4368 (100%)** | **16304 (100%)** | **1859 (100%)** | **7172 (100%)** | **2509 (100%)** | **9132 (100%)** | **9440 (100%)** | **21484 (100%)** |
| **8** | **Pulmonary arterial hypertension** | **11919 (99.7%)** | **30536 (99.7%)** | **4334 (99.2%)** | **16207 (99.4%)** | **1833 (98.6%)** | **7111 (99.1%)** | **2501 (99.7%)** | **9096 (99.6%)** | **9418 (99.8%)** | **21440 (99.8%)** |
| 8 | BMI >40 kg/m2 | 11875 (99.4%) | 30473 (99.5%) | 4195 (96%) | 15975 (98%) | 1760 (94.7%) | 6986 (97.4%) | 2435 (97.1%) | 8989 (98.4%) | 9440 (100%) | 21484 (100%) |
| 8 | Hemoglobin <10 g/dL | 11897 (99.6%) | 30464 (99.5%) | 4276 (97.9%) | 15981 (98%) | 1819 (97.8%) | 7001 (97.6%) | 2457 (97.9%) | 8980 (98.3%) | 9440 (100%) | 21484 (100%) |
| 9 | SBP ≥ 180 mmHg (PARAGON-HF) | 11914 (99.7%) | 30462 (99.5%) | 4305 (98.6%) | 16008 (98.2%) | 1831 (98.5%) | 7030 (98%) | 2474 (98.6%) | 8978 (98.3%) | 9440 (100%) | 21484 (100%) |
| 9 | SBP >150 mmHg and <180 mmHg at baseline, unless the patient is receiving 3 or more antihypertensive drugs (assuming all receive CCBs) | 11939 (99.9%) | 30559 (99.8%) | 4345 (99.5%) | 16165 (99.1%) | 1846 (99.3%) | 7090 (98.9%) | 2499 (99.6%) | 9075 (99.4%) | 9440 (100%) | 21484 (100%) |
| **9** | **SBP <110 mmHg (PARAGON-HF) / <100 mmHg (PARADIGM-HF)** | **10690 (89.5%)** | **27563 (90%)** | **3711 (85%)** | **14175 (86.9%)** | **1590 (85.5%)** | **6298 (87.8%)** | **2121 (84.5%)** | **7877 (86.2%)** | **8569 (90.8%)** | **19686 (91.6%)** |
| 9 | SBP <100 mm Hg or symptomatic hypotension as determined by the investigator at visit 103 or visit 199/201 | Assumed 100% | Assumed 100% | Assumed 100% | Assumed 100% | Assumed 100% | Assumed 100% | Assumed 100% | Assumed 100% | Assumed 100% | Assumed 100% |
| 10 | Use of other investigational drugs | Assumed 100% | Assumed 100% | Assumed 100% | Assumed 100% | Assumed 100% | Assumed 100% | Assumed 100% | Assumed 100% | Assumed 100% | Assumed 100% |
| 11 | History of dilated cardiomyopathy | 11723 (98.1%) | 29852 (97.5%) | 4029 (92.2%) | 15161 (93%) | 1746 (93.9%) | 6793 (94.7%) | 2283 (91%) | 8368 (91.6%) | 9440 (100%) | 21484 (100%) |
| 12 | Evidence of right-sided HF in the absence of left-sided structural heart disease | Assumed 100% | Assumed 100% | Assumed 100% | Assumed 100% | Assumed 100% | Assumed 100% | Assumed 100% | Assumed 100% | -- | -- |
| **13** | **Pericardial constriction/hypertrophic or infiltrative cardiomyopathy (PARAGON-HF)** | **11843 (99.1%)** | **30228 (98.7%)** | **4150 (95%)** | **15492 (95%)** | **1747 (94%)** | **6748 (94.1%)** | **2403 (95.8%)** | **8744 (95.7%)** | -- | -- |
| 14 | Clinically significant congenital heart disease | 11922 (99.8%) | 30508 (99.6%) | 4314 (98.8%) | 16090 (98.7%) | 1832 (98.5%) | 7066 (98.5%) | 2482 (98.9%) | 9024 (98.8%) | 9440 (100%) | 21484 (100%) |
| 15 | Hemodynamically significant valvular disease | Assumed 100% | Assumed 100% | Assumed 100% | Assumed 100% | Assumed 100% | Assumed 100% | Assumed 100% | Assumed 100% | Assumed 100% | Assumed 100% |
| 16 | Stroke or TIA within 3 months prior to index date | 11806 (98.8%) | 30218 (98.7%) | 4335 (99.2%) | 16160 (99.1%) | 1844 (99.2%) | 7113 (99.2%) | 2491 (99.3%) | 9047 (99%) | 9315 (98.7%) | 21171 (98.5%) |
| 17 | Coronary or carotid or valvular heart disease requiring intervention | Assumed 100% | Assumed 100% | Assumed 100% | Assumed 100% | Assumed 100% | Assumed 100% | Assumed 100% | Assumed 100% | Assumed 100% | Assumed 100% |
| 18 | AF and HR >110 | 11918 (99.7%) | 30529 (99.7%) | 4319 (98.9%) | 16145 (99%) | 1841 (99%) | 7100 (99%) | 2478 (98.8%) | 9045 (99%) | 9440 (100%) | 21484 (100%) |
| 19 | CRT | 11084 (92.8%) | 28862 (94.3%) | 4263 (97.6%) | 16015 (98.2%) | 1830 (98.4%) | 7099 (99%) | 2433 (97%) | 8916 (97.6%) | 8651 (91.6%) | 19946 (92.8%) |
| 20 | Previous major transplant | 11935 (99.9%) | 30556 (99.8%) | 4352 (99.6%) | 16220 (99.5%) | 1851 (99.6%) | 7130 (99.4%) | 2501 (99.7%) | 9090 (99.5%) | 9434 (99.9%) | 21466 (99.9%) |
| **21** | **Trial scenario - Mental disorders (PARAGON-HF)** | **11870 (99.3%)** | **30371 (99.2%)** | **4237 (97%)** | **15864 (97.3%)** | **1807 (97.2%)** | **6977 (97.3%)** | **2430 (96.9%)** | **8887 (97.3%)** | -- | -- |
| 21 | Pragmatic scenario - Mental disorders (PARAGON-HF) | 11939 (99.9%) | 30574 (99.9%) | 4348 (99.5%) | 16224 (99.5%) | 1849 (99.5%) | 7134 (99.5%) | 2499 (99.6%) | 9090 (99.5%) | -- | -- |
| 22 | Pancreatic disease within 5 years prior to index date (PARAGON-HF) / Liver disease within 1 year or Crohn within 1 year or Duodenal or gastric ulcers within 3 months (PARADIGM-HF) | 11776 (98.6%) | 30253 (98.8%) | 4361 (99.8%) | 16283 (99.9%) | 1856 (99.8%) | 7160 (99.8%) | 2505 (99.8%) | 9123 (99.9%) | 9271 (98.2%) | 21130 (98.4%) |
| 23 | History of liver disease (PARAGON-HF) | 11915 (99.7%) | 30511 (99.7%) | 4304 (98.5%) | 16105 (98.8%) | 1829 (98.4%) | 7078 (98.7%) | 2475 (98.6%) | 9027 (98.8%) | -- | -- |
| **24** | **eGFR < 30 ml/min/1.73m2** | **11379 (95.2%)** | **29035 (94.8%)** | **4121 (94.3%)** | **15374 (94.3%)** | **1730 (93.1%)** | **6698 (93.4%)** | **2391 (95.3%)** | **8676 (95%)** | **8988 (95.2%)** | **20359 (94.8%)** |
| 24 | eGFR <25 ml/min/1.73 m2 at visit 103 or visit 199/201 | Assumed 100% | Assumed 100% | Assumed 100% | Assumed 100% | Assumed 100% | Assumed 100% | Assumed 100% | Assumed 100% | Assumed 100% | Assumed 100% |
| 24 | eGFR reduction >35% (compared with visit 1) at visit 103 or visit 199/201 | Assumed 100% | Assumed 100% | Assumed 100% | Assumed 100% | Assumed 100% | Assumed 100% | Assumed 100% | Assumed 100% | Assumed 100% | Assumed 100% |
| 25 | Presence of known functionally significant bilateral renal artery stenosis | Assumed 100% | Assumed 100% | Assumed 100% | Assumed 100% | Assumed 100% | Assumed 100% | Assumed 100% | Assumed 100% | Assumed 100% | Assumed 100% |
| 26 | Potassium > 5.2 mmol/l | 11727 (98.1%) | 30189 (98.6%) | 4282 (98%) | 16079 (98.6%) | 1822 (98%) | 7071 (98.6%) | 2460 (98%) | 9008 (98.6%) | 9267 (98.2%) | 21181 (98.6%) |
| 26 | Serum potassium >5.4 mmol/l at visit 103 or visit 199/201 | Assumed 100% | Assumed 100% | Assumed 100% | Assumed 100% | Assumed 100% | Assumed 100% | Assumed 100% | Assumed 100% | Assumed 100% | Assumed 100% |
| 27 | Life expentance < 3 (PARAGON-HF) /5 (PARADIGM-HF) years | Assumed 100% | Assumed 100% | Assumed 100% | Assumed 100% | Assumed 100% | Assumed 100% | Assumed 100% | Assumed 100% | Assumed 100% | Assumed 100% |
| 28 | Noncompliance | Assumed 100% | Assumed 100% | Assumed 100% | Assumed 100% | Assumed 100% | Assumed 100% | Assumed 100% | Assumed 100% | Assumed 100% | Assumed 100% |
| 29 | Drug or alcohol abuse within the past 12 months | 11935 (99.9%) | 30577 (99.9%) | 4343 (99.4%) | 16222 (99.5%) | 1848 (99.4%) | 7129 (99.4%) | 2495 (99.4%) | 9093 (99.6%) | 9440 (100%) | 21484 (100%) |
| 30 | Persons directly involved in the execution of this protocol | Assumed 100% | Assumed 100% | Assumed 100% | Assumed 100% | Assumed 100% | Assumed 100% | Assumed 100% | Assumed 100% | Assumed 100% | Assumed 100% |
| 31 | Malignancy within the past 5 years prior to index (PARAGON-HF) | 11558 (96.7%) | 29246 (95.5%) | 3687 (84.4%) | 13830 (84.8%) | 1569 (84.4%) | 6068 (84.6%) | 2118 (84.4%) | 7762 (85%) | -- | -- |
| 32 | Pregnant or nursing (lactating) women | Assumed 100% | Assumed 100% | Assumed 100% | Assumed 100% | Assumed 100% | Assumed 100% | Assumed 100% | Assumed 100% | Assumed 100% | Assumed 100% |
| 33 | Women of child-bearing potential | Assumed 100% | Assumed 100% | Assumed 100% | Assumed 100% | Assumed 100% | Assumed 100% | Assumed 100% | Assumed 100% | Assumed 100% | Assumed 100% |
|  | Eligible (literal scenario) only exclusion criteria | 7280 (60.9%) | 18156 (59.3%) | 1637 (37.5%) | 6936 (42.5%) | 732 (39.4%) | 3245 (45.2%) | 905 (36.1%) | 3691 (40.4%) | 6375 (67.5%) | 14465 (67.3%) |
|  | Eligible (pragmatic scenario) only exclusion criteria | 10104 (84.6%) | 25823 (84.3%) | 3295 (75.4%) | 12619 (77.4%) | 1364 (73.4%) | 5499 (76.7%) | 1931 (77%) | 7120 (78%) | 8173 (86.6%) | 18703 (87.1%) |
|  | Eligible (literal scenario) | 3944 (33%) | 10681 (35.1%) | 940 (21.5%) | 4087 (25.1%) | 455 (24.5%) | 2063 (28.8%) | 485 (19.3%) | 2024 (22.2%) | 3459 (36.6%) | 8657 (40.6%) |
|  | Eligible (pragmatic scenario) | 7593 (63.5%) | 20957 (68.4%) | 2300 (52.7%) | 9572 (58.7%) | 978 (52.6%) | 4242 (59.1%) | 1322 (52.7%) | 5330 (58.4%) | 6271 (66.4%) | 15627 (72.7%) |

Eligibility estimates reported as numbers and percentages and representing the remaining cohort after applying the respective inclusion/exclusion criteria. The criteria were not ordered and mutually exclusive.

Variables included in the pragmatic scenario in **bold**.

Abbreviations – HF: heart failure; HFpEF: NYHA: New York heart association; NT-proBNP: N-terminal pro-B-type natriuretic peptide; ACEi: Angiotensin converting enzyme inhibitor; ARB: angiotensin receptor blocker; CRT: cardiac resynchronization therapy; SBP: systolic blood pressure; BMI: body mass index; AF: atrial fibrillation; HR: heart rate.

**Supplementary Table 5. Baseline characteristics in eligible vs. non eligible patients in the literal scenario.**

|  | **HFpEF** | | | **HFmrEF** | | | **HFrEF** | | |
| --- | --- | --- | --- | --- | --- | --- | --- | --- | --- |
|  | **Eligible** | **Not eligible** | **p-value** | **Eligible** | **Not eligible** | **p-value** | **Eligible** | **Not eligible** | **p-value** |
| **n** | 1804 | 5368 |  | 1804 | 7330 |  | 8061 | 13423 |  |
| **Demographic/Organizational/Socioeconomic Characteristics** | | | | | | | | | |
| **Sex = Males** | 783 ( 43.4) | 2880 ( 53.7) | <0.001 | 1067 ( 59.1) | 4913 ( 67.0) | <0.001 | 5835 ( 72.4) | 9940 ( 74.1) | 0.008 |
| **Age, median [IQR]** | 81.0 [75.0, 85.0] | 76.0 [67.0, 82.0] | <0.001 | 79.0 [72.0, 84.0] | 73.0 [64.0, 80.0] | <0.001 | 73.0 [65.0, 79.0] | 72.0 [63.0, 80.0] | 0.082 |
| **Follow-up referral = Specialty** | 719 ( 40.8) | 2815 ( 54.1) | <0.001 | 940 ( 53.4) | 4668 ( 65.5) | <0.001 | 6070 ( 77.6) | 10062 ( 77.5) | 0.904 |
| **Follow-up referral HF nurse-led clinic** | 868 ( 50.1) | 2518 ( 49.0) | 0.458 | 936 ( 53.8) | 3771 ( 53.2) | 0.703 | 4335 ( 55.8) | 7425 ( 57.7) | 0.011 |
| **Year of registration** |  |  | 0.075 |  |  | <0.001 |  |  | <0.001 |
| **2000-2005** | 85 ( 4.7) | 206 ( 3.8) |  | 109 ( 6.0) | 304 ( 4.1) |  | 469 ( 5.8) | 641 ( 4.8) |  |
| **2006-2011** | 761 ( 42.2) | 2172 ( 40.5) |  | 740 ( 41.0) | 2733 ( 37.3) |  | 3321 ( 41.2) | 5248 ( 39.1) |  |
| **2012-2016** | 958 ( 53.1) | 2990 ( 55.7) |  | 955 ( 52.9) | 4293 ( 58.6) |  | 4271 ( 53.0) | 7534 ( 56.1) |  |
| **Marital status** |  |  | <0.001 |  |  | <0.001 |  |  | 0.106 |
| **Married** | 815 ( 45.2) | 2570 ( 47.9) |  | 872 ( 48.4) | 3871 ( 52.9) |  | 4074 ( 50.6) | 6963 ( 52.0) |  |
| **Single** | 361 ( 20.0) | 1538 ( 28.7) |  | 445 ( 24.7) | 2237 ( 30.5) |  | 2709 ( 33.7) | 4451 ( 33.2) |  |
| **Widowed** | 627 ( 34.8) | 1257 ( 23.4) |  | 485 ( 26.9) | 1215 ( 16.6) |  | 1261 ( 15.7) | 1985 ( 14.8) |  |
| **Educational level** |  |  | <0.001 |  |  | <0.001 |  |  | 0.030 |
| **Compulsory school** | 948 ( 53.9) | 2356 ( 44.7) |  | 891 ( 50.2) | 2939 ( 40.7) |  | 3521 ( 44.5) | 5620 ( 42.6) |  |
| **Secondary school** | 578 ( 32.9) | 1985 ( 37.7) |  | 626 ( 35.3) | 2962 ( 41.0) |  | 3128 ( 39.5) | 5392 ( 40.9) |  |
| **University** | 233 ( 13.2) | 929 ( 17.6) |  | 258 ( 14.5) | 1320 ( 18.3) |  | 1264 ( 16.0) | 2172 ( 16.5) |  |
| **Income** |  |  | <0.001 |  |  | <0.001 |  |  | 0.016 |
| **Low** | 774 ( 42.9) | 1912 ( 35.6) |  | 662 ( 36.7) | 2223 ( 30.4) |  | 2618 ( 32.5) | 4248 ( 31.7) |  |
| **Medium** | 651 ( 36.1) | 1752 ( 32.7) |  | 698 ( 38.7) | 2359 ( 32.2) |  | 2680 ( 33.3) | 4318 ( 32.2) |  |
| **High** | 378 ( 21.0) | 1701 ( 31.7) |  | 442 ( 24.5) | 2741 ( 37.4) |  | 2746 ( 34.1) | 4833 ( 36.1) |  |
| **Clinical Characteristics** | | | | | | | | | |
| **HF duration ≤ 6 months** | 1119 ( 63.5) | 3436 ( 65.2) | 0.208 | 1164 ( 65.7) | 4205 ( 58.4) | <0.001 | 4277 ( 53.7) | 7702 ( 58.3) | <0.001 |
| **Previous HF hospitalization within 9 months** | 580 ( 32.2) | 1449 ( 27.0) | <0.001 | 615 ( 34.1) | 1724 ( 23.5) | <0.001 | 3778 ( 46.9) | 5376 ( 40.1) | <0.001 |
| **Previous HF hospitalization within 12 months** | 607 ( 33.6) | 1545 ( 28.8) | <0.001 | 651 ( 36.1) | 1854 ( 25.3) | <0.001 | 3967 ( 49.2) | 5659 ( 42.2) | <0.001 |
| **NYHA class** |  |  | <0.001 |  |  | <0.001 |  |  | <0.001 |
| **I** | 0 ( 0.0) | 978 ( 21.9) |  | 0 ( 0.0) | 1328 ( 20.3) |  | 0 ( 0.0) | 2013 ( 16.5) |  |
| **II** | 854 ( 58.3) | 2056 ( 46.0) |  | 995 ( 61.7) | 3483 ( 53.1) |  | 4438 ( 59.6) | 5299 ( 43.5) |  |
| **III** | 588 ( 40.1) | 1349 ( 30.2) |  | 596 ( 36.9) | 1667 ( 25.4) |  | 2922 ( 39.2) | 4550 ( 37.4) |  |
| **IV** | 24 ( 1.6) | 83 ( 1.9) |  | 22 ( 1.4) | 79 ( 1.2) |  | 88 ( 1.2) | 310 ( 2.5) |  |
| **ECG** |  |  | <0.001 |  |  | <0.001 |  |  | <0.001 |
| **Atrial fibrillation** | 842 ( 48.0) | 1978 ( 38.0) |  | 826 ( 46.6) | 2273 ( 31.6) |  | 3135 ( 39.4) | 3375 ( 25.6) |  |
| **PM/other rhythm** | 159 ( 9.1) | 432 ( 8.3) |  | 196 ( 11.1) | 725 ( 10.1) |  | 585 ( 7.3) | 2069 ( 15.7) |  |
| **Sinus rhythm** | 754 ( 43.0) | 2799 ( 53.7) |  | 751 ( 42.4) | 4194 ( 58.3) |  | 4244 ( 53.3) | 7747 ( 58.7) |  |
| **Systolic blood pressure** | 130.0 [120.0, 145.0] | 130.0 [112.0, 140.0] | <0.001 | 130.0 [120.0, 140.0] | 125.0 [110.0, 140.0] | <0.001 | 125.0 [115.0, 140.0] | 120.0 [105.0, 135.0] | <0.001 |
| **Diastolic blood pressure** | 72.0 [65.0, 80.0] | 70.0 [64.0, 80.0] | <0.001 | 75.0 [68.0, 80.0] | 71.0 [65.0, 80.0] | <0.001 | 75.0 [66.0, 80.0] | 70.0 [60.0, 80.0] | <0.001 |
| **Heart rate** | 70.0 [61.0, 80.0] | 70.0 [60.0, 79.0] | 0.055 | 70.0 [62.0, 80.0] | 68.0 [60.0, 78.0] | <0.001 | 70.0 [60.0, 80.0] | 70.0 [60.0, 79.0] | 0.004 |
| **eGFR** |  |  | <0.001 |  |  | <0.001 |  |  | <0.001 |
| **<30 ml/min/1.73m2** | 0 ( 0.0) | 474 ( 9.1) |  | 0 ( 0.0) | 458 ( 6.4) |  | 0 ( 0.0) | 1125 ( 8.5) |  |
| **30-<60 ml/min/1.73m2** | 1064 ( 60.3) | 2097 ( 40.3) |  | 1013 ( 57.0) | 2451 ( 34.1) |  | 3311 ( 41.6) | 5092 ( 38.6) |  |
| **≥60 ml/min/1.73m2** | 701 ( 39.7) | 2638 ( 50.6) |  | 765 ( 43.0) | 4271 ( 59.5) |  | 4641 ( 58.4) | 6979 ( 52.9) |  |
| **Haemoglobin (g/L), median [IQR]** | 130.0 [120.0, 140.0] | 132.0 [120.0, 143.0] | 0.002 | 133.0 [123.0, 143.0] | 136.0 [123.0, 146.0] | <0.001 | 138.0 [127.0, 148.0] | 135.0 [123.0, 147.0] | <0.001 |
| **NT-proBNP (pg/ml), median [IQR]** | 1817.5 [1045.2, 3275.3] | 1161.0 [434.0, 2744.0] | <0.001 | 2071.0 [1120.2, 3813.3] | 1135.0 [437.8, 2629.8] | <0.001 | 2400.0 [1300.0, 4675.5] | 2130.0 [700.0, 5087.0] | <0.001 |
| **Potassium (mEq/L), median [IQR]** | 4.2 [3.9, 4.4] | 4.2 [4.0, 4.5] | <0.001 | 4.2 [4.0, 4.5] | 4.2 [4.0, 4.5] | 0.004 | 4.3 [4.0, 4.5] | 4.3 [4.0, 4.6] | <0.001 |
| **BMI (kg/m2)** |  |  | <0.001 |  |  | <0.001 |  |  | 0.016 |
| **≤30** | 665 ( 67.4) | 2035 ( 67.3) |  | 712 ( 71.2) | 3118 ( 71.4) |  | 3521 ( 73.3) | 6130 ( 75.4) |  |
| **30-40** | 321 ( 32.6) | 804 ( 26.6) |  | 288 ( 28.8) | 1101 ( 25.2) |  | 1166 ( 24.3) | 1845 ( 22.7) |  |
| **>40** | 0 ( 0.0) | 186 ( 6.1) |  | 0 ( 0.0) | 145 ( 3.3) |  | 117 ( 2.4) | 159 ( 2.0) |  |
| **Treatments** | | | | | | | | | |
| **ACEi/ARB** | 1480 ( 82.8) | 4332 ( 81.2) | 0.135 | 1587 ( 88.7) | 6550 ( 89.9) | 0.146 | 7976 (100.0) | 11954 ( 89.6) | <0.001 |
| **MRA** | 540 ( 30.1) | 1515 ( 28.4) | 0.175 | 544 ( 30.4) | 1889 ( 25.9) | <0.001 | 3287 ( 41.0) | 5068 ( 37.9) | <0.001 |
| **Diuretic** | 1799 (100.0) | 3583 ( 67.1) | <0.001 | 1796 (100.0) | 4137 ( 56.7) | <0.001 | 6300 ( 78.4) | 9630 ( 72.0) | <0.001 |
| **Nitrate** | 241 ( 13.4) | 690 ( 12.9) | 0.609 | 282 ( 15.7) | 772 ( 10.6) | <0.001 | 860 ( 10.7) | 1645 ( 12.3) | <0.001 |
| **Antiplatelet** | 590 ( 33.2) | 1916 ( 36.4) | 0.018 | 665 ( 37.3) | 3249 ( 45.0) | <0.001 | 3028 ( 37.9) | 6388 ( 48.3) | <0.001 |
| **Anticoagulant** | 984 ( 54.9) | 2468 ( 46.1) | <0.001 | 1001 ( 55.7) | 3169 ( 43.4) | <0.001 | 4151 ( 51.6) | 5794 ( 43.3) | <0.001 |
| **Statin** | 761 ( 42.3) | 2399 ( 44.8) | 0.074 | 896 ( 49.9) | 3900 ( 53.4) | 0.009 | 3933 ( 49.0) | 7336 ( 54.8) | <0.001 |
| **Beta-blocker** | 1492 ( 83.0) | 4389 ( 81.9) | 0.338 | 1571 ( 87.4) | 6464 ( 88.4) | 0.254 | 8047 (100.0) | 11924 ( 89.0) | <0.001 |
| **Digoxin** | 309 ( 17.2) | 689 ( 12.9) | <0.001 | 326 ( 18.1) | 824 ( 11.3) | <0.001 | 1485 ( 18.5) | 1652 ( 12.3) | <0.001 |
| **HF device** |  |  | <0.001 |  |  | <0.001 |  |  | <0.001 |
| **CRT-P** | 0 ( 0.0) | 35 ( 0.7) |  | 0 ( 0.0) | 117 ( 1.6) |  | 0 ( 0.0) | 630 ( 4.8) |  |
| **CRT-D** | 0 ( 0.0) | 38 ( 0.8) |  | 0 ( 0.0) | 101 ( 1.4) |  | 0 ( 0.0) | 908 ( 6.9) |  |
| **ICD** | 4 ( 0.2) | 64 ( 1.3) |  | 20 ( 1.2) | 127 ( 1.8) |  | 347 ( 4.4) | 562 ( 4.3) |  |
| **Comorbidities** | | | | | | | | | |
| **Anemia** | 555 ( 32.0) | 1620 ( 31.9) | 0.945 | 505 ( 29.0) | 1930 ( 27.8) | 0.327 | 1830 ( 23.6) | 3862 ( 30.1) | <0.001 |
| **Smoking** |  |  | <0.001 |  |  | <0.001 |  |  | 0.038 |
| **Current** | 67 ( 5.4) | 354 ( 9.2) |  | 99 ( 7.6) | 651 ( 12.0) |  | 857 ( 13.5) | 1321 ( 12.6) |  |
| **Former** | 489 ( 39.2) | 1630 ( 42.2) |  | 573 ( 44.1) | 2396 ( 44.3) |  | 2924 ( 46.0) | 5020 ( 48.0) |  |
| **Never** | 691 ( 55.4) | 1878 ( 48.6) |  | 626 ( 48.2) | 2367 ( 43.7) |  | 2569 ( 40.5) | 4123 ( 39.4) |  |
| **Hypertension** | 1289 ( 72.6) | 3197 ( 60.7) | <0.001 | 1171 ( 66.3) | 3674 ( 51.1) | <0.001 | 4060 ( 51.7) | 6106 ( 46.8) | <0.001 |
| **Diabetes** | 446 ( 24.9) | 1254 ( 23.5) | 0.252 | 449 ( 25.0) | 1565 ( 21.4) | 0.001 | 1899 ( 23.6) | 3228 ( 24.2) | 0.367 |
| **Ischemic heart disease** | 759 ( 42.1) | 2301 ( 42.9) | 0.575 | 937 ( 51.9) | 3878 ( 52.9) | 0.478 | 3569 ( 44.3) | 7918 ( 59.0) | <0.001 |
| **Myocardial infarction** | 312 ( 17.3) | 1089 ( 20.3) | 0.006 | 424 ( 23.5) | 2291 ( 31.3) | <0.001 | 1755 ( 21.8) | 4712 ( 35.1) | <0.001 |
| **Coronary revascularization** | 404 ( 22.4) | 1210 ( 22.5) | 0.923 | 535 ( 29.7) | 2412 ( 32.9) | 0.009 | 2101 ( 26.1) | 4947 ( 36.9) | <0.001 |
| **Peripheral vascular disease** | 163 ( 9.0) | 520 ( 9.7) | 0.442 | 232 ( 12.9) | 680 ( 9.3) | <0.001 | 758 ( 9.4) | 1414 ( 10.5) | 0.008 |
| **Stroke/TIA** | 283 ( 15.7) | 717 ( 13.4) | 0.015 | 293 ( 16.2) | 919 ( 12.5) | <0.001 | 909 ( 11.3) | 1907 ( 14.2) | <0.001 |
| **Atrial fibrillation** | 1175 ( 65.6) | 2830 ( 53.2) | <0.001 | 1133 ( 63.3) | 3413 ( 46.9) | <0.001 | 4086 ( 50.9) | 5669 ( 42.5) | <0.001 |
| **Valvular disease** | 555 ( 30.8) | 1415 ( 26.4) | <0.001 | 483 ( 26.8) | 1477 ( 20.2) | <0.001 | 1308 ( 16.2) | 2444 ( 18.2) | <0.001 |
| **COPD** | 269 ( 14.9) | 800 ( 14.9) | 1.000 | 271 ( 15.0) | 921 ( 12.6) | 0.006 | 1008 ( 12.5) | 1810 ( 13.5) | 0.041 |
| **Liver disease (within 1 year)** | 0 ( 0.0) | 94 ( 1.8) | <0.001 | 0 ( 0.0) | 107 ( 1.5) | <0.001 | 0 ( 0.0) | 292 ( 2.2) | <0.001 |
| **Malignancy (within 5 years)** | 0 ( 0.0) | 1104 ( 20.6) | <0.001 | 0 ( 0.0) | 1372 ( 18.7) | <0.001 | 1046 ( 13.0) | 1880 ( 14.0) | 0.035 |
| **Pancreatic disease (within 1 year)** | 0 ( 0.0) | 100 ( 1.9) | <0.001 | 0 ( 0.0) | 111 ( 1.5) | <0.001 | 0 ( 0.0) | 311 ( 2.3) | <0.001 |
| **Mental disease (within 1 year)** | 0 ( 0.0) | 195 ( 3.6) | <0.001 | 0 ( 0.0) | 247 ( 3.4) | <0.001 | 286 ( 3.5) | 525 ( 3.9) | 0.188 |

Abbreviations – HF: heart failure; NYHA: New York heart association; NT-proBNP: N-terminal pro-B-type natriuretic peptide; ACEi: Angiotensin converting enzyme inhibitor; ARB: angiotensin receptor blocker; MRA: mineralocorticoid receptor antagonist; ICD: implantable cardioverter defibrillator; CRT-D: cardiac resynchronization therapy-defibrillator; CRT-P: cardiac resynchronization therapy-pacemaker; COPD: chronic obstructive pulmonary disease; IQR: interquartile range; BMI: body mass index; PARAGON-HF The Prospective Comparison of ARNI [angiotensin receptor neprilysin inhibitor] with ARB [angiotensin- receptor blockers] Global Outcomes in HF with Preserved Ejection Fraction trial; PARADIGM-HF: Prospective Comparison of ARNI With ACEI to Determine Impact on Global Mortality and Morbidity in Heart Failure; EF: ejection fraction; eGFR: estimated glomerular filtration rate; TIA:Transient Ischemic Attack; ECG: electrocardiogram

**Supplementary Table 6. Outcome analysis according to eligibility status**

| **Pragmatic scenario** | | | | **Literal scenario** | | | |
| --- | --- | --- | --- | --- | --- | --- | --- |
| **EF<50%** | | | | | | |  |
|  | **Eligible** | **Non-eligible** | **P** | **Eligible** | **Non-eligible** | **P** |  |
| **All-cause mortality** | 11.8 (11.5-12.1) | 9.5 (9.1-9.8) | <0.001 | 10.4 (10.0-10.7) | 11.2 (10.9-11.5) | <0.001 |  |
| **CV mortality** | 7.5 (7.3-7.7) | 5.8 (5.5-6.0) | <0.001 | 6.5 (6.2-6.7) | 7.0 (6.8-7.2) | 0.003 |  |
| **First HF hospitalization** | 12.2 (11.9-12.6) | 9.1 (8.8-9.5) | <0.001 | 11.5 (11.1-11.9) | 10.8 (10.5-11.0) | 0.003 |  |
| **CV mortality + first HF hospitalization** | 16.4 (16.1-16.8) | 12.1 (11.7-12.5) | <0.001 | 15.0 (14.6-15.5) | 14.5 (14.2-14.9) | 0.082 |  |
| **Non-CV mortality** | 4.3 (4.2-4.5) | 3.7 (3.5-3.9) | <0.001 | 3.9 (3.7-4.1) | 4.2 (4.1-4.4) | 0.017 |  |
| **EF≥40%** | | | | | | |  |
| **All-cause mortality** | 12.2 (11.7-12.6) | 8.9 (8.5-9.3) | <0.001 | 13.5 (12.8-14.2) | 9.7 (9.4-10.0) | <0.001 |  |
| **CV mortality** | 7.0 (6.7-7.3) | 4.9 (4.7-5.2) | <0.001 | 8.2 (7.7-8.7) | 5.4 (5.1-5.6) | <0.001 |  |
| **First HF hospitalization** | 9.2 (8.8-9.6) | 6.9 (6.5-7.2) | <0.001 | 10.8 (10.1-11.5) | 7.3 (7.0-7.6) | <0.001 |  |
| **CV mortality + first HF hospitalization** | 13.4 (12.9-13.9) | 9.5 (9.1-9.9) | <0.001 | 15.5 (14.7-16.3) | 10.4 (10.1-10.7) | <0.001 |  |
| **Non-CV mortality** | 5.2 (4.9-5.5) | 3.9 (3.7-4.2) | <0.001 | 5.3 (4.9-5.7) | 4.4 (4.2-4.6) | <0.001 |  |
| **EF≥50%** | | | | | | |  |
| **All-cause mortality** | 13.0 (12.3-13.6) | 9.7 (9.2-10.3) | <0.001 | 13.8 (12.8-14.8) | 10.6 (10.1-11.1) | <0.001 |  |
| **CV mortality** | 7.3 (6.8-7.8) | 5.4 (5.0-5.9) | <0.001 | 8.1 (7.3-8.9) | 5.8 (5.4-6.2) | <0.001 |  |
| **First HF hospitalization** | 9.6 (9.0-10.2) | 7.4 (6.9-7.9) | <0.001 | 10.2 (9.3-11.2) | 7.9 (7.5-8.4) | <0.001 |  |
| **CV mortality + first HF hospitalization** | 13.8 (13.1-14.6) | 10.3 (9.7-10.9) | <0.001 | 14.7 (13.7-15.9) | 11.2 (10.7-11.8) | <0.001 |  |
| **Non-CV mortality** | 5.7 (5.3-6.2) | 4.3 (3.9-4.7) | <0.001 | 5.7 (5.1-6.3) | 4.8 (4.5-5.1) | 0.009 |  |
| **EF=40-49%** | | | | | | |  |
| **All-cause mortality** | 11.5 (11.0-12.1) | 8.3 (7.8-8.7) | <0.001 | 13.2 (12.3-14.1) | 9.1 (8.7-9.5) | <0.001 |  |
| **CV mortality** | 6.7 (6.3-7.2) | 4.6 (4.2-4.9) | <0.001 | 8.3 (7.5-9.0) | 5.0 (4.8-5.3) | <0.001 |  |
| **First HF hospitalization** | 8.9 (8.4-9.4) | 6.4 (6.0-6.9) | <0.001 | 11.3 (10.4-12.3) | 6.8 (6.5-7.2) | <0.001 |  |
| **CV mortality + first HF hospitalization** | 13.0 (12.4-13.7) | 8.9 (8.4-9.4) | <0.001 | 16.2 (15.0-17.3) | 9.8 (9.4-10.2) | <0.001 |  |
| **Non-CV mortality** | 4.8 (4.5-5.2) | 3.7 (3.4-4.0) | <0.001 | 4.9 (4.4-5.5) | 4.1 (3.8-4.3) | 0.006 |  |
| **EF<40%** | | | | | | |  |
| **All-cause mortality** | 11.9 (11.6-12.2) | 10.2 (9.8-10.6) | <0.001 | 9.8 (9.5-10.2) | 12.4 (12.1-12.7) | <0.001 |  |
| **CV mortality** | 7.7 (7.5-8.0) | 6.5 (6.2-6.8) | <0.001 | 6.1 (5.8-6.4) | 8.1 (7.9-8.4) | <0.001 |  |
| **First HF hospitalization** | 13.4 (13.0-13.8) | 11.0 (10.5-11.5) | <0.001 | 11.5 (11.1-12.0) | 13.3 (12.9-13.7) | <0.001 |  |
| **CV mortality + first HF hospitalization** | 17.6 (17.1-18.0) | 14.3 (13.8-14.9) | <0.001 | 14.8 (14.3-15.3) | 17.6 (17.2-18.1) | <0.001 |  |
| **Non-CV mortality** | 4.2 (4.0-4.4) | 3.7 (3.5-4.0) | 0.003 | 3.7 (3.5-3.9) | 4.3 (4.1-4.5) | <0.001 |  |

Event rates reported as *100 patient-years

Cardiovascular death has been defined according to the ICD-10 codes I00-99 as main cause of death. Non-cardiovascular death has been defined according to any ICD-10 other than I00-99 as main cause of death.

Abbreviations - EF: ejection fraction; CV: cardiovascular; HF: heart failure
